# Hormonal treatments for endometriosis: The endocrine background

**DOI:** 10.1007/s11154-021-09666-w

**Published:** 2021-08-17

**Authors:** Silvia Vannuccini, Sara Clemenza, Margherita Rossi, Felice Petraglia

**Affiliations:** Obstetrics and Gynecology, Department of Experimental, Clinical and Biomedical Sciences, University of Florence, Careggi University Hospital, Florence, Italy

**Keywords:** Activin, AMH, Aromatase inhibitors, CRH, Dienogest endometriosis, Estrogens, Progesterone-resistance, GnRH agonist, GnRH antagonist, Hormones, Inflammation, Inhibin, Progestin, SERMs, SPRMs, Stress

## Abstract

Endometriosis is a benign uterine disorder characterized by menstrual pain and infertility, deeply affecting women’s health. It is a chronic disease and requires a long term management. Hormonal drugs are currently the most used for the medical treatment and are based on the endocrine pathogenetic aspects. Estrogen-dependency and progesterone-resistance are the key events which cause the ectopic implantation of endometrial cells, decreasing apoptosis and increasing oxidative stress, inflammation and neuroangiogenesis. Endometriotic cells express AMH, TGF-related growth factors (inhibin, activin, follistatin) CRH and stress related peptides. Endocrine and inflammatory changes explain pain and infertility, and the systemic comorbidities described in these patients, such as autoimmune (thyroiditis, arthritis, allergies), inflammatory (gastrointestinal/urinary diseases) and mental health disorders.

The hormonal treatment of endometriosis aims to block of menstruation through an inhibition of hypothalamus-pituitary-ovary axis or by causing a pseudodecidualization with consequent amenorrhea, impairing the progression of endometriotic implants. GnRH agonists and antagonists are effective on endometriosis by acting on pituitary-ovarian function. Progestins are mostly used for long term treatments (dienogest, NETA, MPA) and act on multiple sites of action. Combined oral contraceptives are also used for reducing endometriosis symptoms by inhibiting ovarian function. Clinical trials are currently going on selective progesterone receptor modulators, selective estrogen receptor modulators and aromatase inhibitors. Nowadays, all these hormonal drugs are considered the first-line treatment for women with endometriosis to improve their symptoms, to postpone surgery or to prevent post-surgical disease recurrence. This review aims to provide a comprehensive state-of-the-art on the current and future hormonal treatments for endometriosis, exploring the endocrine background of the disease.

## Introduction

Endometriosis is a chronic disease characterized by the presence of endometrium-like tissue outside the uterine cavity, affecting women of reproductive age with pelvic pain and infertility [1]. The prevalence ranges between 2 and 10% of women in reproductive age, 30–50% among infertile women, and 5 to 21% among women with severe pelvic pain [2]. However, the true prevalence is uncertain, because estimates vary widely among population samples and diagnostic approaches [3].

The pathophysiology of endometriosis is still a matter of investigation, but endocrine and inflammatory backgrounds are well characterized, recognizing an estrogen-dependency [4] and a progesterone-resistance [5]. The main mechanisms involved in the ectopic location of endometrial cells include retrograde menstruation, vascular and lymphatic spread and/or metaplasia/stem cells. The most accepted theory is the retrograde menstruation, according to which menstrual endometrial fragments migrate through the fallopian tubes to the peritoneal cavity, where they implant, proliferate and invade pelvic peritoneum. The back flow of endometrial cells into the pelvis is physiologic, resulting apoptosis/autophagy and cell-mediated immunity the scavenger system for eliminating these cells, while in endometriotic patients hormonal influences and genetic/epigenetic factors determine an impairment of these mechanisms, promoting cell survival, proliferation and peritoneal invasion. [6]. Increased estrogen receptors activity, estrogen production in endometriotic lesions and progesterone-resistance are the determinants of impaired apoptosis, reduced immune function and increased inflammation [7–9]. Thus, endometriotic cells attach, penetrate and invade the peritoneum, determining growth of lesions which undergo cyclic bleeding with repeated tissue injury and repair [10], neoangiogenesis [11] and neurogenesis [12]. Fibroblast–myofibroblast transdifferentiation contributes to collagen production and fibrogenesis [13], with entrapment of nerve fibers which, associated with chronic inflammation, explain pain symptoms.

According to the location of the lesions three phenotypes of endometriosis are recognized: ovarian endometriomas (OMA) (the most common, characterized by typical chocolate cysts) superficial peritoneal endometriosis (SUP) and deep infiltrating endometriosis (DIE) (the most severe forms developing deeper than 5 mm under the peritoneal surface also infiltrating the muscularis propria of bladder or bowel) [1]. In addition, extraperitoneal locations are described, i.e. pleura, diaphragm or umbilicus [14] and 30% of cases endometriosis are associated to adenomyosis (infiltration by endometrial stroma and glands into the myometrium) [15, 16].

The most common symptoms of endometriosis are menstruation-related pain, i.e. dysmenorrhea, dyspareunia, dysuria and dyschezia, and noncyclic pelvic pain may also occurs in these patients. Since these symptoms are not specific to endometriosis and may be signs of other gynecological or non-gynecological conditions, misdiagnosis or a significant delay in endometriosis identification is frequently reported [17].

Painful symptoms and infertility are also associated with psychological stress, low self-esteem, and depression impairing physical, mental, and social well-being [18] and reducing quality of life (QoL) [19]. Therefore, these patients, other than hypothalamus-pituitary-ovary axis (HPO) changes, show also an impairment of hypothalamus–pituitary–adrenal axis (HPA) and thyroid function, and comorbidities associated with inflammation and immune dysfunction.

In the last two decades an increased diagnosis/incidence of endometriosis has been observed and its chronic and progressive nature determines a relevant impact in a lifelong perspective among these patients. In the past, surgery was considered the definitive treatment, but recent evidences showed that it does not solve the pathogenetic mechanisms and patients need a long-term management. Treatment goals are pain control and fertility improvement maximizing the use of medical treatment, but also postsurgical prevention of symptoms and lesions recurrence, in order to avoid repeated surgical procedures [20, 21]. In fact, surgery in women with endometriosis is associated with the risk of urological, intestinal, vascular and neurological complications and pain may recur or persist in case of incomplete excision of endometriosis lesions [22, 23]. Presently, the medical therapy is considered the first-line treatment for most of women with endometriosis to improve their symptoms, but also to plan the most adequate timing of surgery or assisted reproductive technologies (ART) treatment, or to prevent post-surgical disease recurrence [1, 24, 25]. The choice of the most appropriate therapy is based on the intensity of pain, age, desire to conceive, but also on the impact of the disease on QoL on each patient [26].

Currently, hormonal treatments are the most effective drugs for the treatment of endometriosis and are based on the pathogenic mechanisms involved in the disease. The goal is to stop cyclic menstruation: by blocking ovarian estrogen secretion or by causing a pseudopregnancy state [21]. The endocrine background provides the rationale for the current and future hormonal drugs for treating women with endometriosis.

## Endocrine changes in endometriosis

### HPO axis hormones

#### FSH and LH

No significant difference in terms of serum follicle-stimulating hormone (FSH) and luteinizing hormone (LH) levels were found between women with endometriosis and controls. However, some FSHR and LHR single nucleotide polymorphisms (SNPs) have been observed in endometriosis patients [27]. FSHR 680Ser-Ser/GG genotype and ‘‘GG/307Ala680Ser’’ haplotype were more frequently found in fertile women with endometriosis, while the presence of "GA/307Ala680Asn" haplotype lowers the likelihood of disease onset and progression [28, 29]. Besides, the SS (680 Ser/Ser) or AA (307 Ala/Ala) genotype are associated with a reduced risk to develop stage 3–4 endometriosis compared to the stage 1–2 endometriosis [30]. FSHR 680Asn/Asn induces aromatase activity resulting in higher estrogens levels and proliferation of endometriotic lesions [31]. Among LHR SNPs, a polymorphic insertion in exon 1 of LH receptor (LHR) gene (insLQ) is common in women with endometriosis and infertility, and it is thought to boost LHR activity, by decreasing the half maximal effective concentration and increasing the cell surface expression [32].

#### Estrogens and ERs

**Fig. 1:**
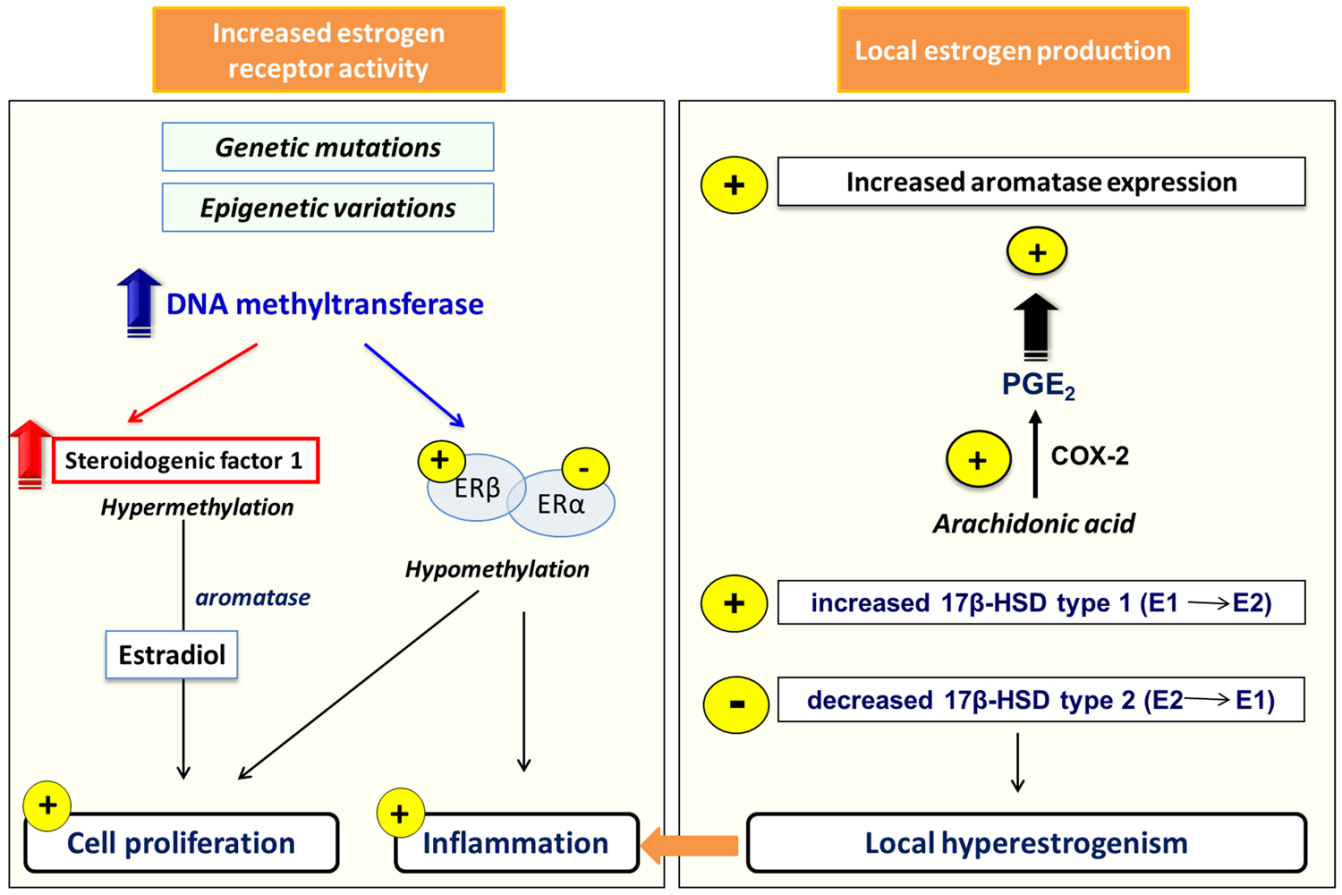
Estrogen receptors activity and local estrogens production in endometriosis. 17β-HSD, 17β-hydroxysteroid dehydrogenase. E2, estradiol. E3, estrone

Although serum estrogen levels in endometriosis patients are not significantly different from those in healthy women, it is clear that estrogen-mediated alterations play a role in the etiology of endometriosis: an estrogen dominance is caused by to a local estrogens synthesis and to an increased ERs activity in endometriotic cells (Fig. 1). Estrogens are a significant biologic driver of chronic inflammation, promoting endometriotic cell survival and lesion progression. Clear data show that endometriotic tissue expresses the entire set of steroidogenic genes, including aromatase, allowing to local de-novo estradiol (E2) production. [33]. Local E2 levels are increased in endometriosis due to upregulation of the aromatase gene CYP19A1 [34] and reduction of 17-hydroxysteroid dehydrogenase type 2 (17HSD2), which normally (induced by P4) converts E2 to the less potent estrone [35, 36]. The endometriotic stromal cells are epigenetically dysregulated and express steroidogenic proteins and enzymes such as steroidogenic acute regulatory protein (STAR) and convert the precursor molecule cholesterol to E2. The fundamental event in E2 synthesis is the recruitment of the nuclear receptor steroidogenic factor-1 (SF-1) to the promoters of these steroidogenic genes is the key event for E2 synthesis [37].A feed-forward loop connects hyperestrogenic stimulation with inflammation: the overexpression of cyclooxygenase 2 (COX2) and CYP19A1 increases local production of prostaglandins and estrogen, causing a vicious circle [38]. The overproduction of estradiol in endometriosis drives ERβ signaling to support endometriotic tissue survival and inflammation.

In terms of alterations in ERs activity, an overexpression of ERβ and a downregulation of ERα [39, 40] have been observed in endometriosis. Changes in promoter methylation may be a cause for the increased ERβ/ERα ratio in endometriotic cells, since regions of the ERα promoter become hypermethylated leading to a decreased expression, whereas a CpG island in the ERβ promoter becomes hypomethylated causing an increased expression [41, 42] (Fig. 1). When compared to controls, ERα levels are higher in the eutopic endometrium of women with endometriosis, resulting in enhanced estrogenic activity and proliferation, which impacts endometrial function. ERβ expression is unchanged in eutopic endometrium of women with endometriosis, although an increased ERβ/ERα ratio has been observed [43]. An important role is played by steroid receptor coactivators (SRCs) [44], and expression profiling of SRCs in endometriotic lesions identified SRC-1 as the predominant SRC [45]. Despite a drop in overall SRC-1, levels of a truncated form are increased in animal and human models. This new isoform of SRC-1 *in vitro* decreases tumor necrosis factor alpha (TNFα)-mediated apoptosis in endometriotic cells, promoting increased cell survival and invasion and reflecting the *in vivo* disease pathophysiology [45]. In addition, SRC-1 isoform and ERβ may mediate a synergistic role in promoting cell survival in endometriosis [46].

Estrogens have a major role for endometriotic tissue attachment to peritoneum, lesion survival, production of inflammatory substances (metalloproteinases, cytokines, or prostaglandins and growth factors) and angiogenesis. ERβ triggers pathways that enhance lesion survival, remodel pelvic peritoneal tissue, and produce inflammatory substances, which stimulate nociceptors in pelvic tissues, leading to pain [47]. The pathologic levels of local estradiol biosynthesis seems to induce also decrease of apoptosis in endometriotic stromal and epithelial cells compared with eutopic endometrial tissues [48–50]. Estrogens also mediate immune system dysregulation in endometriotic lesion. Peritoneal fluid macrophages from women with endometriosis upregulate the expression of ERβ and in a mouse model of endometriosis E2 treatment increases the macrophages present in lesions as well as the expression of macrophage migration factor [51, 52].

#### Progesterone and PRs

Circulating progesterone (P4) levels are similar to those found in healthy women. In endometriosis a typical dysregulation of progesterone signaling and an endometrial tissue inability to appropriately respond to progesterone exposure identifies the condition of progesterone resistance. It manifests in endometriosis as failed induction of PRs activation, or P4 target gene transcription in presence of bioavailable P4 [53]. Progesterone resistance has been well-established in both the endometriotic lesions and eutopic endometrium of women with endometriosis [54]. Since P4 signaling is required to counteract E2-induced proliferation and to promote decidualization [55], the loss of P4-responsiveness leads to both an increased growth of endometriotic lesions and to a non-receptive endometrium [33, 56].

**Fig. 2:**
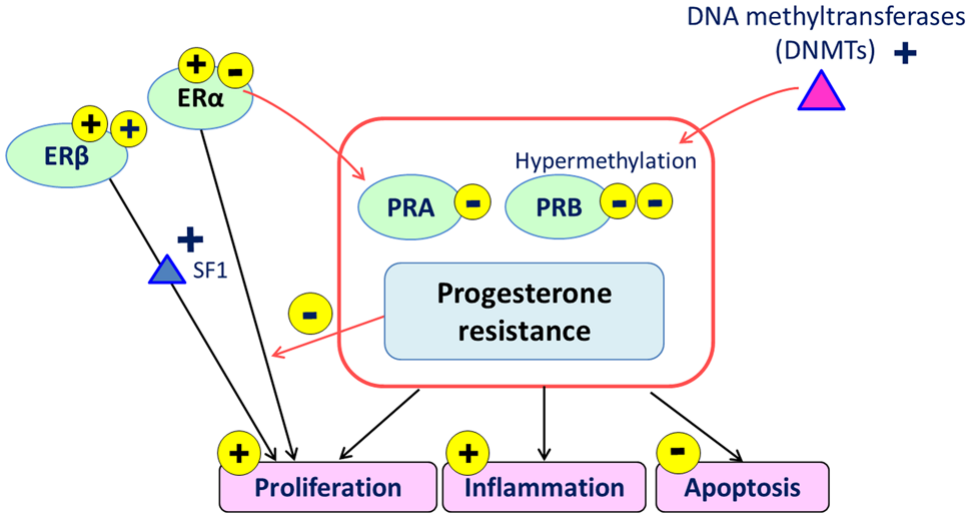
The mechanisms of progesterone-resistance in endometriosis

Changes in the expression of the nuclear PR isoforms PR-A and PR-B, of steroid receptor coactivators, and of multiple downstream effectors in endometriotic lesions and eutopic endometrium from women with endometriosis represent the molecular cause of progesterone resistance. (Fig. 2). The concept of progesterone resistance was suggested by the finding that in endometriotic lesions of PR-B was undetectable and PR-A was markedly lower compared with normal endometrium [57]. Promoter hypermethylation and microRNA dysregulation were suggested to be potential mechanisms for PR-B loss in endometriosis. In fact, aberrations in genetic and epigenetic regulation of PRs and their targets have been demonstrated [27]. Progesterone receptor (PR) gene polymorphism may also promote the susceptibility to endometriosis [58]. Among polymorphisms described in the PR gene of patients with endometriosis, the PROGINS polymorphism affects ligand-binding and downstream signaling in the cellular context of endometriosis, and is involved in progesterone resistance [59, 60].

Furthermore, in endometriotic tissue, P4 does not induce epithelial 17β-HSD-2 expression [35], an enzyme which in normal endometrium induces the expression of the enzyme 17β-hydroxysteroid dehydrogenase type 2 (17β-HSD-2), which metabolizes the biologically active estrogen E_2_ to estrone. This additional deficiency, when combined to excessive estradiol production due to aberrant aromatase activity, contributes to the abnormally high estradiol activity in endometriosis. P4 also influences inflammatory pathways, suppressing the signaling of members of the nuclear factor kappa light-chain-enhancer of activated B cells (NF-kB) family of proteins in endometrial cells. This signaling network has been implicated in endometriosis as a factor leading to the establishment and maintenance of endometriosis implants [61].

#### Inhibin, activin and follistatin

Inhibins and activins belong to the transforming growth factors (TGF)-ß superfamily and are involved in the regulation of cellular proliferation, differentiation and apoptosis of endometrial cells. Inhibin A, inhibin B and activin A are detectable in the peritoneal fluid of women with pelvic endometriosis and cultured endometriotic cells expressed the mRNA of inhibin, ßA-, ßB-subunits, and activin receptors types II and IIB [62]. Both α and ß A subunits are expressed in glands and stroma of OMA, and their dimers inhibin A and activin A are more concentrated in the cystic fluid than in peritoneal fluid [63], suggesting that they may be contributing to both implantation defects in eutopic endometrium and to the development of ectopic locations of endometriosis [64]. Indeed, in cultured human endometrial stromal cells from women with endometriosis, activin A increases IL-6 and IL-8 secretion [65, 66]. An impaired expression of OMA and endometrial cripto (activin receptor antagonist), and follistatin (activin-binding protein) indicates an impaired activin pathway in endometriosis [67]. Furthermore, nodal, a growth factor highly expressed in high turnover tissues and acting through SMAD proteins, has shown only subtle changes in endometriosis, differentiating the high proliferation of endometriosis cells from malignancies [68]. Serum activin A and follistatin are not significantly altered in SUP or DIE phenotypes and have limited diagnostic accuracy in the diagnosis of OMA [69].

#### Anti-mullerian hormone (AMH)

AMH is a dimeric glycoprotein belonging to the transforming growth factor-β superfamily and besides its functional role in the ovary, it reflects the number of preantral follicles that comprise the oocyte pool, thus serum AMH level serves as a marker of ovarian reserve [70].

A significantly decrease of serum AMH levels was reported in women with OMA compared with age-matched fertile controls [71]. Conversely, the adverse effect of surgical removal of OMA on ovarian reserve parameters including AMH levels is well recognized [72–74]. Thus, the impact of endometriosis and OMA per se on the ovarian reserve is still subject to controversy [72]. Moreover, infertility patients with endometriosis showed lower AMH level compared to women with a primary diagnosis of male factor infertility [75]. This conclusion is confirmed by recent data [76] indicating that serum AMH level in infertile patients with OMA is significantly lower than in the control group and patients with bilateral OMAs have lower AMH levels than those with unilateral OMA. Moreover, patients with previous cystectomy had a considerably lower mean serum AMH level than individuals with OMA who had never had surgery. These findings suggest that OMA per se is associated with reduced ovarian reserve, and laparoscopic cystectomy can further exert significant damage on ovarian reserve. Anyhow, patients with OMA experience a progressive decline in serum AMH levels, which is faster than that in healthy women [77]. The contrasting observation that AMH levels are not diminished in women with endometriosis, including those with presence of uni- or bilateral OMAs unless they had had previous OMA surgery, was based on data from women undergoing surgery without information on infertility, thus biasing the results [72].

The mechanism of OMA inducing ovarian reserve damage is still elusive. The inflammatory response to the endometriosis implants [78] may cause microscopic alterations of the follicular and vascular patterns. Moreover, the compression of surrounding ovarian cortex by the cyst could hamper circulation and cause follicle loss [79]. However, further studies are needed to elucidate the mechanisms of ovarian reserve damage induced by OMA. There are limited data regarding the effect of SUP or DIE, without OMA, on ovarian reserve [80], showing that the effect of extraovarian endometriosis on ovarian reserve is less pronounced than that of OMA.

AMH is also produced by eutopic and ectopic endometriotic cells and it secreted in peritoneal fluid [81]. The treatments with AMH *in vitro* decreases the proliferative activity and increases the intracellular signal of apoptosis, suggesting a role of AMH in the pathogenesis of the disease [82–84].

## Other endocrine aspects

### HPA axis and stress hormones

Endometriosis-related pain and infertility elicit a stress response: from one side, infertility provokes family issues and fear of frustrating social expectations [85, 86], on the other pelvic pain causes sexual dysfunction and work absenteeism [87], all of which contribute to increase anxiety and chronic stress. Since endometriosis is also surrounded by apprehension about the disease progression, the long-term health risks and the prospect of having children may be additional sources of stress [87–89]. Furthermore, women with endometriosis experience a delay of 4 to 7 years from first presentation of symptoms to the diagnosis [90, 91], which may further enhance the levels of stress perceived by the patient. Women with endometriosis and severe endometriosis-related pain (dysmenorrhea, pelvic pain, dyspareunia) usually present with very high scores of perceived stress [92]. However, if on one hand surgical treatment of symptomatic women reduces perceived stress, women undergoing to multiple surgeries reported high stress scores impairing the QoL [93]. In fact, endometriosis has a significantly negative impact on health-related QoL scores and the factors involved are mainly linked to pain symptoms [94]. A recent study by Marki et al. reported that both physical pain symptoms and emotional regulation difficulties, the latter being mediated by psychological stress, reduced health-related QoL of women with endometriosis [95]. Furthermore, other aspects, such as self-confidence, body esteem, and emotional self-efficacy, play a role in the psychological health and stress perception, being impaired among women with endometriosis[96].

**Fig. 3:**
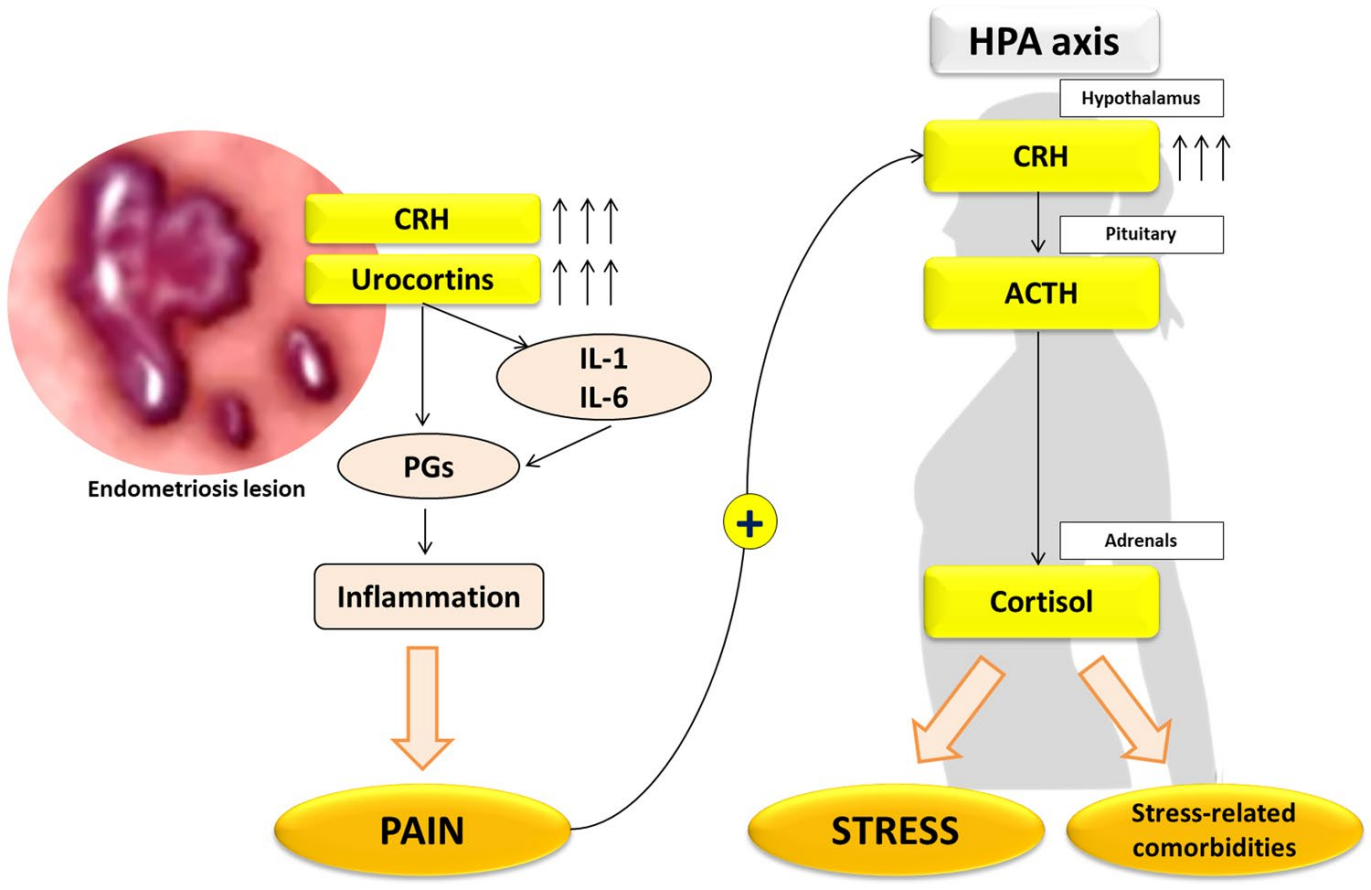
Hypothalamus–pituitary–adrenal (HPA) axis and stress hormones in endometriosis

A dysfunction of HPA axis is found in patients with endometriosis [97] (Fig. 3) and it is related to an attenuated cortisol response, a condition known as burnout. A paradoxical hypocortisolism like an adrenal fatigue [98] may exacerbate painful symptoms by reducing the endogenous analgesia associated with stress (stress-induced analgesia) [99] [100]. Supporting this assumption, a blunted early morning cortisol response to CRH test was associated with greater menstrual and non-menstrual pain in endometriosis [101]. Low salivary cortisol levels and a high degree of perceived stress were found to be associated with poor QoL in patients with endometriosis and chronic pelvic pain [102], as well as salivary hypocortisolism, which was linked to infertility and dyspareunia but not dysmenorrhea [103]. On the other hand, higher hair cortisol levels were found in patients with endometriosis compared to healthy women of similar age, parity, education level and BMI [104]. Moreover, increased serum cortisol levels were detected in infertile women with endometriosis, especially in those with advanced stage of disease [105]. Interestingly, physical and psychological interventions have been shown to normalize salivary cortisol levels of women with endometriosis-related chronic pain [106].

CRH and urocortin (Ucn) are also produced by endometrium and locally act modulating tissue differentiation (decidualization of endometrial stroma, embryo implantation and maintenance of pregnancy) and inflammation [107] (Fig. 3). Eutopic endometrium highly express CRH, CRHR type 1 and 2, as well as urocortin mRNA and protein [108], thus suggesting that a deranged CRH and Ucn mRNA expression associated with an impaired CRH-R1 activity may affect the process of decidualization and contribute to infertility in these patients. In fact, cultured endometrial cells from endometriotic patients have a reduced decidualization capacity, reducing the secretion of prolactin, CRH and Ucn [108]. The most intense immunostaining for CRH and Ucn is observed in DIE lesions, with an increased expression of CRH-R1 and R2 and inflammatory enzymes PLA2G2A and COX2 [109]. Since CRH and Ucn significantly increase COX2 expression (effect was reversed by the CRH-R2 antagonist astressin) and endometriotic tissue expresses both Ucn 2 and Ucn 3 (which modulate TNF-α and IL-4 secretion) an involvement of this stress pathway in inflammation is suggested [110].

High levels of CRH-binding protein in peritoneal fluid from women with endometriosis than in controls has been observed, suggesting possible changes also in circulating levels [62]. Plasma urocortin levels are twice as high in women with OMA, and levels are significantly higher in the cystic fluid of OMA than in the peritoneal fluid and plasma [111]. Besides, the preoperative blood testing of Ucn among symptomatic women undergoing surgery for suspect of endometriosis showed that confirmed cases had higher plasma Ucn levels compared to patients with no lesions and elevated plasma Ucn1 levels are found among all endometriosis phenotypes. However, no cutoff could accurately distinguish endometriosis from other pathological conditions, thus it is not useful [112].

### Thyroid hormones

Autoimmune thyroid disorders are frequently found in endometriosis patients suggesting a pathogenic association between these two conditions [113–115]. A relationship between endometriosis and the presence of thyroid autoantibodies is found, leading either to hypothyroidism or hyperthyroidism. The relative risk of endometriosis is significantly increased in women tested positive for thyroperoxidase (TPO) antibodies [114], similarly a high prevalence of anti-TSHR antibodies, pathognomonic of Grave’s disease, is shown in patients with endometriosis [115]. It is not clear whether these antibodies or thyroid hormones play a role in the pathogenesis of endometriosis. A microarray analysis of mild versus severe endometriosis confirmed a potential involvement of thyroid hormone homeostasis and metabolism in the pathophysiology of endometriosis [116].

A recent ex vivo study [117] on thyroid transcripts in patients with endometriosis described an overexpression of TSHR and a decreased biosynthesis of T3 and an accumulation of T4 in ectopic endometrium. The direct stimulation of estrogen receptors on endometrial cells by thyroid hormones was suggested to cause cell proliferation. In fact, *in vitro* studies demonstrated that TSH activates the proliferation of all endometriotic and control cells, T4 has a specific proliferative effect on epithelial and stromal ectopic endometrial cells, whereas T3 only acts on epithelial cells. In addition, thyroid hormones cause ROS production by ectopic endometrial cells, that may favor, in turn, endometriotic cell proliferation [118].

Thyroid hormones may also contribute to the pathogenesis of endometriosis by modulating the immune response, as they can active neutrophils and macrophages to locally promote a proinflammatory environment [119]. Therefore, an increase of serum TSH or of T4 could be hypothesized as a participating factor for endometriosis development and progression. A more sever chronic pelvic pain and disease score in endometriotic patients with a thyroid disorder confirm that endometriosis should be carefully monitored in patients with comorbid thyroid disease [117].

## Clinical implications: pain, infertility and systemic comorbidities in endometriosis

Endometriosis is a heterogeneous disease also in terms of clinical presentation. Common symptoms include dysmenorrhea and non-menstrual pelvic pain, which may develop into chronic pelvic pain [17]. with a relevant impact on daily life [120]. Other endometriosis-related pain are dyspareunia, dyschezia, and dysuria, usually associated with DIE lesions [121, 122]. According to the anatomical involvement of bowel, patients may alternate constipation and diarrhea, dischezia or blood in the stool (in particular perimenstrually) [122, 123] or when urinary tract is affected, recurrent dysuria, cyclic macrohaematuria or interstitial cystitis are observed [124]. Chest and shoulder pain should be considered suspecting diaphragmatic endometriosis [125], whereas endometriosis in the ileo-caecal or peri-appendiceal region has been significantly associated to abdominal pain, nausea, vomiting and diarrhea [126, 127].

Regarding the physiopathology of endometriosis-related pain, nociceptive (including inflammatory), neuropathic and a combination of these mechanisms are involved [128], under the influence of hormonal aberrations, stress, inflammation, and the interplay between the peripheral and central nervous systems [129–131]. Neurogenic factors, such as brain-derived neurotrophic factor (BDNF) and nerve growth factor (NGF) are reported to be overexpressed in the peritoneal fluid and in endometriotic lesions of affected women [132]. Neurotrophic factors are also responsive to estrogens, prostaglandin and cytokine and stimulate the growth and sensitization of sensory nerve fiber terminals [133, 134], particularly in DIE, presenting high nerve fibers density [135]. The development of a vicious cycle characterized by nociceptor sensitization and local neo-neurogenesis, triggered by inflammatory and immune mediators, is observed in endometriosis [136]. Endometriotic lesions themselves send noxious signals to dorsal root spinal cord neurons and activate spinal microglia to maintain pain stimuli, resulting in a central sensitization [137]. In fact, a number of central changes are observed: alterations in the behavioral and central response to noxious stimulation, changes in brain structure, altered activity of both the HPA and the autonomic nervous system and psychological distress [131], with larger volume in regions involved in pain modulation and endocrine function regulation [137–139]. Indeed, chronic pain and stress experienced by patients with endometriosis might cause multiple psychiatric diseases (Fig. 3) and the somatoform disorder is the most common [140]. Anxiety and depression traits, and a higher tendency of pain catastrophizing are commonly present in endometriosis patients and can amplify the perception of pain [141, 142]. Another frequently present, but often neglected, symptom in women with endometriosis is chronic fatigue, although the exact mechanism remains not fully understood [143].

Women with endometriosis show a higher prevalence of systemic comorbidities has been shown, even though it is still unclear whether a common endocrine, immune and inflammatory background predispose to the development of those conditions or a high levels of perceived stress [144, 145]. An increased risk of inflammatory bowel diseases (Chron’s disease, ulcerative colitis) [146] allergic manifestations [sinus allergic rhinitis, and food allergy) [147], autoimmune diseases (systemic lupus erythematosus, rheumatoid arthritis, Sjogren’s syndrome, multiple sclerosis, fibromyalgia) are more likely to be diagnosed in women with endometriosis, also underlying a neuroendocrine–immune imbalance [148–153].

Infertility is the other major symptom of endometriosis, even though a diagnosis of endometriosis does not always imply infertility. Endometriosis is identified in approximately 30% of women in infertile couples [154]. The disease adversely affects fertility by different mechanisms acting at the level of pelvic cavity, ovary and uterus [155]. Pelvic cavity is an hostile environment because the chronic inflammatory changes in the peritoneal fluid and the distortion of normal anatomy of the fallopian tubes hindering tubo-ovarian contact and affecting sperm-oocyte interaction; ovary produces low quality oocytes, impaired folliculogenesis, and luteal function, with ovarian reserve reduced by OMA and/or by surgery. Besides, in endometriosis uterus itself has an altered endometrial receptivity mainly due to local growth factors changes (integrin, LIF, activin, CRH), to hormonal aberrations (ER and PR) [9] and to dysperistalsis of myometrium, due to the association to adenomyosis [156, 157]. However, the evidences supporting the impairment of endometrial receptivity in endometriosis are still controversial. The endometrial chronic inflammation, together with progesterone resistance, estrogen dominance, aberrant cell signaling pathways and reduced expression of key homeostatic proteins in women with endometriosis, are disruptive to endometrial receptivity [158]. On the contrary, data from *in vitro* fertilization (IVF) and egg donations, other than basic data regarding the transcriptomic signature of the endometrium, seem to indicate that endometrial receptivity gene signature during the window of implantation is similar between infertile women with and without endometriosis, suggesting a major effected played by embryo and oocyte quality more than to the endometrial factor itself [159].

## Endocrine background of hormonal treatments for endometriosis

**Fig. 4:**
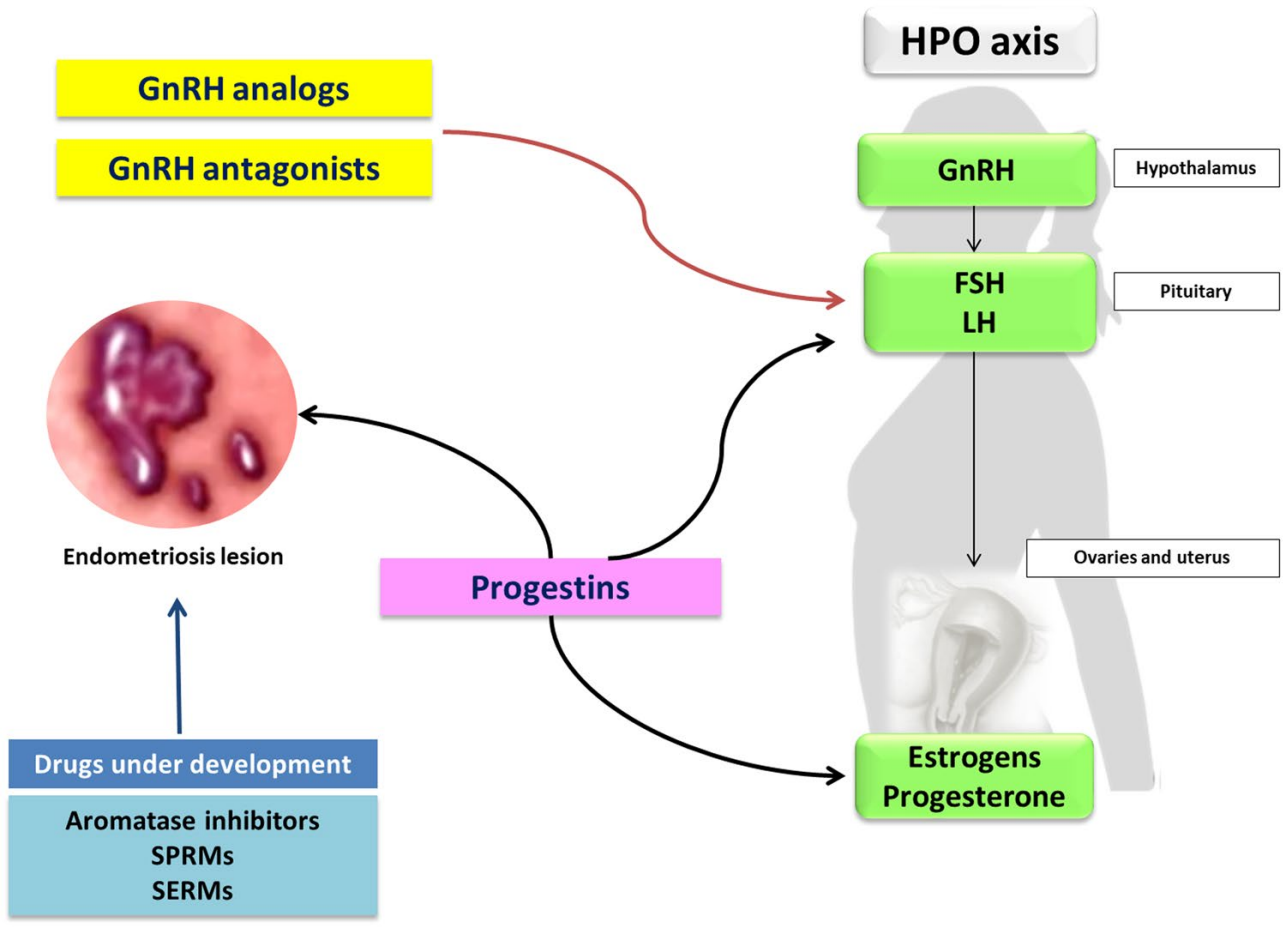
Hormonal targets of currently used drugs for endometriosis

Hormonal therapies are the most common used for treating women with endometriosis. The goal is to block menstruations by causing a state of iatrogenic menopause or pseudopregnancy. Current hormonal medical therapy does not cure definitively the disease, but it is able to control pain symptoms in order to prevent or postpone surgery and to long term manage the disease [21, 160]. First-line hormonal therapies include progestins, while second-line therapy are represented by GnRH agonists (GnRH-a) and antagonists. The off-label use of combined oral contraceptives (COCs) is common. New hormonal drugs (aromatase inhibitors, selective estrogen receptor modulators (SERM), selective progesterone receptor modulators (SPRM)) are under investigation for the treatment of endometriosis (Fig. 4).

### Gonadotropin releasing hormone agonists (GnRH-a)

GnRH-a (goserelin, leuprolide, nafarelin, buserelin, and triptorelin) are labelled drugs used since the ‘90 s to treat endometriosis. They bind to the GnRH receptors and, during the first 10 days of treatment, stimulate the pituitary to produce LH and FSH [161]. Subsequently, the prolonged and continuous exposure to these agents cause downregulation of the GnRH receptors, thus decreasing LH and FSH levels and suppressing estrogen ovarian production (Fig. 4). The induced hypoestrogenism with subsequent amenorrhoic state leads to the regression of endometriotic lesions [162]. Several trials have shown that GnRH-a improved endometriosis-associated pain [163–166] and a meta-analysis of 41 trials comparing the use of GnRH-agonists at different doses, regimens and routes of administration, reports that GnRH-a are more effective than placebo and as effective as other progestins for relieving pain [167]. In particular, the administration of GnRH-a for a period of 3 to 6 months prior to ART in women with endometriosis may increase the odds of clinical pregnancy by fourfold [168].

However, treatment with GnRH-a is associated with significant hypoestrogenic side effects, including amenorrhea, vasomotor symptoms, sleep disturbance, urogenital atrophy, and accelerated bone loss. Therefore, GnRH-a should be used carefully in adolescents since these women may not have reached maximum bone density [169]. The addition of add-back therapy (low-dose COCs, estrogen or progestins alone, bisphosphonates, tibolone or raloxifene), may reduce these adverse effects, without reducing the efficacy of pain relief. With the addition of add-back therapy, the administration of GnRH-a, which was initially limited to 6 months, is allowed for longer time [170]. Some clinical trials and cohort studies have demonstrated that a GnRH-a plus steroid add-back therapy can be effective from 30 months to up to 10 years [171, 172].

### GnRH antagonists

GnRH antagonists suppress gonadotropin hormone production, by competing with endogenous GnRH for its pituitary receptors (Fig. 4). Contrary to GnRH-a, antagonists do not provoke the initial flare-up phase and cause a rapid onset of the therapeutic effect [21]. They have also the advantage of being administered orally because of its non-peptide structure which avoids the gastrointestinal proteolysis.

Elagolix, a short-acting GnRH antagonist, has been recently approved in USA for the management of moderate to severe pain associated with endometriosis [21]. Compared to the classic GnRHa, elagolix, by blocking endogenous GnRH signalling, causes a dose-related suppression of LH and FSH, and a consequent modulation of estradiol levels. Thus, it provides relief of endometriosis-related pain avoiding severe hypoestrogenism [21]. The FDA approved elagolix for the treatment of endometriosis related pain following the results of two multicenter, double-blind, randomized, phase 3 trials [173] which compared two distinct doses of elagolix (150 mg once daily or 200 mg twice daily) with placebo. In both trials, during the 6 months treatment, elagolix dramatically reduced dysmenorrhea and non-menstrual pelvic discomfort. Also in women who still menstruated, a lower proportion of menstrual period days with moderate or severe dysmenorrhea compared with placebo was shown, indicating pain reduction despite continued menses [174]. Positive results were found in two phase 3 extension studies [175], which evaluated long-term efficacy and safety of elagolix for 12 months decreasing dysmenorrhea, nonmenstrual pelvic pain and dyspareunia. Moreover, treatment with elagolix improves QoL [175, 176], decreasing the use of analgesic agents [175] and fatigue levels [177]. Although it inhibits ovarian function in a dose-dependent manner, elagolix especially the higher dose, causes hypoestrogenic side effect, such as hot flash, decrease in BMD and increase in serum lipid levels. Based on those observations, two ongoing Phase III trials are currently examining the safety and efficacy of both elagolix alone and elagolix plus E2 and NETA for the treatment and management of moderate to severe pain in premenopausal women with endometriosis over a 24-months period (NCT03343067 and NCT03213457). Further studies are also needed to evaluate the drug effects on ovarian function, as a number of pregnancies have been reported while taking elagolix; as a result,, patients should use non-hormonal contraceptive systems during the treatment [178, 179].

Relugolix and linzagolix are the two new oral GnRH antagonists, in an advanced stage of clinical development for the management of pain associated with endometriosis [180, 181]. A Phase 2, multicenter, randomized, double-blind, placebo-controlled study on oral administration of relugolix for 12 weeks demonstrated efficacy in alleviating endometriosis-associated pain in a dose–response manner with some adverse events (hot flush, heavy menstrual bleeding, and irregular menstruation, and bone mineral density decrease). However, oral relugolix at the dose of 40 mg was generally well tolerated and showed similar efficacy and safety compared with those of leuprorelin [180]. A Phase 3 extension trial is ongoing aiming to assess the tong-term efficacy and safety of relugolix 40 mg once daily co-administered with low-dose estradiol and norethindrone acetate on endometriosis-associated pain. A Phase 2b, double-blind, placebo-controlled, dose-ranging study with linzagolix, has been performed in women surgically confirmed endometriosis and moderate-to-severe endometriosis-associated pain [181]. Doses ≥ 75 mg resulted in a significantly greater proportion of responders for overall pelvic pain, dysmenorrhea and non-menstrual pelvic pain after 12 and 24 weeks treatment. Serum estradiol was suppressed, QoL improved, and the rate of amenorrhea increased in a dose-dependent fashion. Also mean BMD loss (spine) increased in a dose-dependent manner and was < 1% at 24 weeks at doses of 50 and 75 mg and up to 2.6% for 200 mg. The most frequently reported adverse events related to the trial treatments were hot flushes and headaches [181].

### Progestins

Progestins are compounds with multiple actions on PRs: decreased secretion of FSH and LH, anovulation, relatively hypoestrogenic state and amenorrhea that help suppressing endometriosis and preventing dysmenorrhea. Moreover, they have antiestrogenic effect causing endometrial pseudodecidualisation, inhibit inflammatory response, provoke apoptosis of endometriotic cells, reduce oxidative stress, inhibit angiogenesis and suppress expression of matrix metalloproteinases [5, 26] (Fig. 4). All these mechanisms induced by progestins have a beneficial effect on the progression of endometriosis and associated-pain.

According to the ESHRE guidelines, progestins are considered as a first choice for the treatment of endometriosis [182], because they are as effective in reducing scores and pain as GnRH agonists, and have a lower cost and a lower incidence of adverse effects.

Progestins can be administered by an oral, intramuscular, subcutaneous, or intrauterine route [183]. The progestins most commonly used for the treatment of endometriosis-related pain include dienogest (DNG) norethindrone acetate (NETA) and medroxyprogesterone acetate (MPA) [169, 184]. DNG is approved in Europe, Japan, Australia and Singapore, while NETA and MPA are currently approved by the USA Food and Drug Administration (FDA). Alternative progestin treatment options include gestrinone, desogestrel, danazol, the etonogestrel implant and the levonorgestrel intrauterine system (LNG-IUS). Side effects of progestins include irregular uterine bleeding/spotting, weight gain, mood changes (eg, depression), and bone loss (specific to long-term use of depot MPA). Although these side effects are frequent, they rarely cause therapy abandonment. Overall, progestins are safe and about two thirds of patients are satisfied with their use for symptomatic endometriosis [185].

#### Dienogest

DNG, a 19-nortestosterone derivative, is the most recent progestin available and labelled for endometriosis and according to a number of evidences it substantially improves endometriosis-related pain symptoms in long term treatment [186]. Both surgically and clinically diagnosed patients described comparable pain reduction, as well as women with or without prior treatment [187]. Compared to danazol, MPA and goserelin, DNG is the most efficient alternative to treat pelvic pain associated to endometriosis [188]. Furthermore, no effects on bone mineral density were reported compared to treatment with leuprolide, maintaining a stable bone turnover [189]. Regarding the effects of DNG according to different endometriosis phenotypes, it causes a significant reduction in both diameter and volume of OMA, whereas the ovarian reserve appears to be preserved [190]. Among women with ultrasound identified OMAs followed up for 12 months, DNG reduced the volume of OMAs up to 76% from the initial size. Besides, a reduction of 74.05% for dysmenorrhea, 42.71% for dyspareunia and 48.91% for chronic pelvic pain were observed [191]. Furthermore, DNG alone has been shown to be superior to COCs, containing DNG, to reduce the size of OMAs [192]. A recent study showed that in women with OMA DNG reduces the size of ovarian cysts, effective in reducing endometriosis related symptoms both after 6 and 12 months of treatment and well tolerated [193].

DNG also appears to be effective in controlling pain caused by rectovaginal endometriosis [194], bladder endometriosis [195, 196] and DIE [197]. In a prospective cohort study including 30 women with a sonographic diagnosis of DIE (intestinal and posterior fornix) treated with DNG for 12 months, the treatment was effective to control symptoms of pain related to DIE (dysmenorrhea, dyspareunia, dischezia), improving QoL, even without reducing the volume of DIE nodules [198].

Patients treated with DNG have also shown an improvement of sexual functioning [194] and QoL [187, 199]. Among Asian women, DNG therapy decreased Endometriosis Health Profile-30 (EHP-30) scores in all assessed domains, especially the "pain" domain was improved in 78.4% of patients. Both surgically and clinically diagnosed patients described comparable pain reduction [200]. In randomized controlled trial among Chinese women with endometriosis, DNG for 24 weeks provided significantly greater reduction in endometriosis-associated pelvic pain than placebo, and maintained or enhanced efficacy after 28 weeks of additional treatment [201].

Long-term (60-month) treatment effectively reduced endometriosis-associated pain and avoided pain recurrence post-surgery without sever adverse effects [202, 203], especially on bone mineral density (BMD)[189]. Therefore, its use as first-line therapy for long-term management of debilitating and chronic endometriosis-associated pain represents an interesting option. Regarding efficacy and tolerability, a large study conducted in Korea showed that satisfaction scores were mostly favorable. The most frequently reported side effects are abnormal uterine bleeding (4.1%), weight gain (2.5%) and headache (1.2%). The number of patients with favorable bleeding patterns was observed to increase as the duration of treatment increases, till amenorrhea [204].

DNG is an effective treatment also as postoperative treatment in order to reduce recurrences, to avoid re-interventions and to control pain symptoms. DNG is as effective and tolerable as GnRH agonist and add–back therapy using 17b-estradiol and NETA for 6 months for the prevention of pelvic pain recurrence after laparoscopic surgery for endometriosis [205]. A prospective cohort study on women undergoing to surgery for OMA, receiving postoperative medical treatment with DNG for 24 months no cases of OMA recurrence were found [206]. In case of recurrent OMA after surgery, DNG therapy early after recurrence appears to be viable for reducing the risk of repeated surgery, given that after 24 months of treatment with DNG, a reduction of size [186] and complete resolution of recurrent OMA was achieved in 57.1% [207].

#### Norethindrone acetate (NETA)

NETA, another 19-nortestosterone derivative, is effective on pain relief in women with endometriosis. NETA has strong progestogenic effects and a androgenic activity, that may cause side effects due to its residual androgenic activity (weight gain, acne, and seborrhea) [208]. The continuous administration of NETA (5 mg/day) for the treatment of endometriosis is approved by the US FDA. Low-dose NETA 2.5 mg/day orally is considered an effective, tolerable and inexpensive first choice for symptomatic rectovaginal endometriosis, significantly decreasing VAS scores for dysmenorrhea and dyspareunia [209]. A pilot study on women with bowel endometriosis showed that low dose oral NETA determined a significant improvement in the intensity of chronic pelvic pain, deep dyspareunia, dyschezia and the disappearance of symptoms related to the menstrual cycle (dysmenorrhea, constipation during the menstrual cycle, diarrhea during the menstrual cycle and cyclical rectal bleeding) [210]. Recently, a long term study 5-year therapy with NETA [2.5 mg/day up to 5 mg/day) is safe and well tolerated by women with rectovaginal endometriosis, who were satisfied or very satisfied in 68.8% of cases. Due to its low cost and good pharmacological profile, it may represent a good candidate for long-term treatment for endometriosis [211]. Low dose of NETA was also found to have less side effects, such as unscheduled bleeding, compared to extended-cycle COCs, despite the same effectiveness in terms of pain control [212]. The comparison between low dose NETA and DNG as first line drug used in new diagnosed endometriosis women showed that treatment was well tolerated by 58% of NETA users compared with 80% of DNG users [208]. However, in a subpopulation of symptomatic women with rectovaginal endometriosis “NETA "resistant”, who had pain persistence, DNG was effective in treating pain and improving QoL [213].

#### Medroxyprogesterone acetate (MPA)

MPA is a 17-OH progesterone derivative, available as oral formulation or depot formulation, which can be administered intramuscularly and subcutaneously every 3 months. MPA appeared to be more effective than placebo [214] and as effective as danazol [215] and GnRH-agonists [216, 217] in reducing endometriosis-related pain. In particular, depot MPA (dMPA) reduces pain as effectively as leuprolide and improves quality of life and productivity. The major source of concern regarding continuous use of depot MPA is the loss of BMD with an increased risk of fracture, due to estrogens deficiency. Therefore, the FDA have suggested that it should be administrated only if other methods are unsuitable or unacceptable, and have limited its maximum use to 2 years [218]. On the contrary, the American College of Obstetricians and Gynecologists support the use of dMPA as current longitudinal and cross-sectional evidence suggests the recovery of BMD after discontinuation of dMPA, and, considering the modest increase in the risk of fracture, benefits of dMPA use surpasses the risks.

#### Danazol

Danazol is a derivative of 17α-ethynyl testosterone and since 1971 is approved by FDA to treat endometriosis. Its mechanisms of action include inhibition of pituitary gonadotropin secretion, direct inhibition of ovarian enzymes responsible for estrogen production, modulation of immunological function, suppression of cell proliferation and inhibition of endometriotic implant growth [170, 219]. Danazol is effective in treating endometriosis-related pain [220] and its efficacy seems to persist also after the discontinuation of therapy [215]. However, its use is limited by the androgenic-type adverse effects such as seborrhea, hypertrichosis, weight gain, HDL levels decrease, and LDL levels increase [170]. Danazol is typically given orally (400 to 800 mg/day). Good efficacy and better tolerability has been reported with danazol-loaded intrauterine device [221] and with off-label vaginal administration (200 mg/day) [222, 223], particularly for women with DIE and rectovaginal endometriosis [224]. A significant reduction of painful symptoms in patients with DIE was observed, with less recurrences and a decreased volume of endometriosis lesions [223]. Furthermore, long-standing use of vaginal danazol suppositories resulted in favourable control of postoperative pelvic pain associated with pelvic endometriosis without significant adverse side effects [225]. Low-dose vaginal danazol (200 mg per day for 6 months) is effective also for the treatment of pain in recurrent endometriosis after surgery for severe disease, with reduction of VAS pain intensity [226]. With low doses and vaginal route of administration, side- effects are seldom observed, and lipid parameters and liver function are reported to be unaltered.

#### Other progestins

##### Desogestrel

Desogestrel (DSG) (75 mg/day) is an effective, safe and low cost therapy for endometriosis related pain [227, 228] with a good satisfaction rate and causing also a significant improvement in QoL. DSG treatment of women with symptomatic rectovaginal endometriosis induced volume size reduction and improvement of gastrointestinal symptoms, chronic pelvic pain, and deep dyspareunia. At 12‐month follow up, the rate of satisfied patients was higher in those treated with the desogestrel‐only pill compared to those on sequential estro-progestin pill [229]. DSG resulted effective also in A significant improvement of both pelvic pain and dysmenorrhea after 6-months treatment in endometriosis recurrence. Breakthrough bleeding is the main adverse effect reported during DSG treatment [227].

##### Levonorgestrel intrauterine device (LNG-IUS)

The effect of the LNG-IUS on endometriosis has been assessed in several RCTs. LNG induces endometrial glandular atrophy and decidual transformation of the stroma, reduces endometrial cell proliferation and increases apoptotic activity. After the first year of use, a 70–90% reduction in menstrual blood loss is observed. The LNG-IUS has proven effective in relieving pelvic pain symptoms caused by peritoneal and rectovaginal endometriosis and in reducing the risk of recurrence of dysmenorrhea after conservative surgery [230]. In fact, LNG-IUS use after surgery was associated with a significantly lower dysmenorrhea recurrence rate compared to ﻿with expectant management [231–233]. Dyspareunia and dysmenorrhea were clearly reduced after 12-months of treatment with few adverse events and very low discontinuation rate [234]. A recent study evaluated the efficacy of LNG-IUS versus DNG treatment compared to no post-operative therapy after laparoscopic surgery for endometriosis. At 6 and 12 months, the median pain scores in treatment groups were significantly lower and both treatments had significantly lower recurrence rate than control group (3.8% and 9.7%, respectively, vs 32.5%). In addition, patients with LNG-IUS showed lower rate of discontinuation, suggesting that LNG-IUS is effective for postoperative pain control and for preventing recurrence [235]. However, no or limited effect was observed in preventing OMA recurrence. In fact, in a randomized clinical trial including 80 patients with OMA undergoing laparoscopic cystectomy followed by six cycles of GnRH-a, and then allocated to LNG-IUS insertion or not for 30 months, LNG-IUS was able to control pain symptoms but it was not effective for preventing OMA recurrence [236]. However, a recent meta-analysis on the efficacy of different hormonal regimens for the prevention of OMA recurrence in women who have undergone conservative surgery showed that among cohort studies LNG-IUS ranked highest [237].

##### Gestrinone

In a meta-analysis including two small studies, treatment with gestrinone resulted effective in reduction of pain [214]. However, the use of gestrinone for endometriosis is limited due to its side effects. In fact, because of its androgenic, anti-estrogenic and anti-progestogenic properties, it may cause acne, seborrhea, hirsutism, weight gain, liver dysfunction and osteoporosis [238].

##### Etonogestrel-releasing subdermal implant (ENG- implant)

Few data are available also on the use of the etonogestrel-releasing subdermal implant (ENG- implant) for the treatment of women with endometriosis, resulting effective in decreasing dyspareunia, dysmenorrhea and nonmenstrual pelvic pain [239, 240]. A recent study evaluating the efficacy of ENG-implant versus the 52-mg LNG-IUS in the control of endometriosis-associated pelvic pain showed that both contraceptives improved significantly pelvic pain, dysmenorrhea, and health-related quality of life in endometriosis [241].

### Combined oral contraceptives (COCs)

COCs are currently used off label for the treatment of endometriosis, however they are commonly used as empirical therapy for women with suspect of endometriosis, without a confirmed surgical diagnosis of the disease [242]. ESHRE guidelines classifies as Grade B the COCs prescription to reduce dyspareunia, dysmenorrhea and non-menstrual pain. On the other hand, Grade C evidence has been provided to COCs continuous use in women suffering endometriosis-associated pain [182]. The advantages of using COCs for the treatment of endometriosis include the good tolerability as well as the low costs, but they contain estrogens. COCs reduce menstrual flow, cause decidualisation of endometriotic implants and decrease cell proliferation [243]. Ovarian function is inhibited as well as the metabolism of arachidonic acid to prostaglandins, resulting effective in reducing pelvic pain and menstrual cramps. Although COCs are widely used in clinical practice since decades, given their effectiveness for dysmenorrhea, high level evidence of their effectiveness for the treatment of endometriosis does not exist.

Only two trials [244, 245], both conducted in Japan, compared COCs with placebo in women with endometriosis. In these studies, COCs treatment was associated with an improvement in dysmenorrhea, cyclical non-menstrual pain, dyspareunia and dyschezia. However, the formulation of COCs used in these studies (ethinylestradiol 35 mcg + norethisterone 1 mg in cyclic regimen and ethinylestradiol 20 mcg + drospirenone 3 mg in flexible regimen) may not be readily available globally and it is unknown if different formulations may have different effects [246].

In a recent systematic review about patient response to medical therapies for endometriosis [247], the rate of patients experiencing pain symptoms at the end of treatment was higher with COC, vaginal ring and patch compared to GnRH-a or progestins. The observation that about 50% of patients have partial or no improvement in symptoms of endometriosis under COCs [248] and about 70% of women had used multiple COCs for relief of pain and over 40% had been prescribed between 3 and 10 different COCs [249] support the conclusion that this treatment is not completely effective [250]. Despite the low dose COCs (20–30 μg is equivalent to 4 to 6 times the physiologic dose of estrogens) and, given ER and PR alterations in endometriosis, the administration of COCs may result in estrogen dominance in the presence of progesterone resistance [250]. Studies also showed an increased risk of endometriosis in past users of COCs [251].

Some studies showed that COCs prevent and reduce frequency and severity of recurrent dysmenorrhea and relapse of endometriosis after surgery [252–256]. The continuous use of COCs after conservative surgery is more beneficial than the cyclic use [253, 256, 257]. However, COCs after previous surgery has similar [258] or less efficacy in pain relief than GnRH-a [259]. In conclusions, despite their wide use in clinical practice, further research is needed to fully evaluate the role of COCs in the management of endometriosis-related pain.

### Drugs under development for endometriosis

#### Selective progesterone receptor modulators (SPRM)

SPRMs are progesterone receptor ligands that act as tissue-selective progesterone agonist, antagonist, or partial agonist/antagonist on various progesterone target tissues. Although SPRMs inhibit the ovulation, they are not associated with the systemic effects of estrogen deprivation as estradiol secretion is not affected and circulating levels of estradiol remain in the physiological range. Furthermore, SPRMs inhibit the endometrial proliferation, suppress endometrial bleeding through a direct effect on endometrial blood vessels, and reduce endometrial prostaglandin production in a tissue-specific manner [21] (Fig. 4). Therefore, a potential good efficacy of SPRMs on endometriosis was suggested [260–262], but no SPRMs are used in clinical practice.

Ulipristal acetate (UPA), telapristone acetate, vilaprisan and tanaproget are SPRMs which were proposed for the treatment of endometriosis [263, 264]. SPRMs are generally well tolerated. Common adverse effects are headache, abdominal pain, nausea, dizziness, and heavy menstrual bleeding. Mifepristone and asoprisnil were the most studied SPRMs. Mifepristone-induced regression of endometriotic lesions has been variable and appears to be dependent on the duration of treatment [265, 266]. A small prospective open-label trial suggested the possible efficacy of mifepristone for endometriosis-associated pain [265]. Similar results were found in a phase II/III trial; however, 3,4% of patients reported a significant increase in hepatic transaminases [267]. In a randomized placebo-controlled trial, asoprisnil caused a higher decrease of dysmenorrhea among women affected by endometriosis compared to placebo [268]. The effect of UPA was assessed on endometriosis lesions and symptoms in women treated over a 27-month study period prior to surgery. In 58% of cases progesterone receptor modulator-associated endometrial changes (PAECs) were observed in both eutopic endometrium and ectopic lesions; those cases reported all pain reduction and amenorrhea [269]. However, there are insufficient data to permit firm conclusions about their safety and effectiveness [21].

#### Selective estrogen receptor modulators (SERMs)

SERMs bind to estrogen receptors (ER-α and ER-β) in target cells acting as ER agonist in some tissues and ER antagonist in others (Fig. 4), and therefore they have been proposed for the treatment of endometriosis and are under investigation. Raloxifene (RLX), a common drug approved for the prevention and treatment of osteoporosis, has estrogenic effects in bone and antiestrogenic effects in endometrium and breast tissue [162]. Tested in animal studies RLX induces regression of endometriosis implant [270, 271]. In a double-blind prospective study [272], patients with endometriosis-related pelvic pain following surgical treatment were randomly assigned to RLX or placebo for 6 months. However, this study was halted prematurely because women in RLX group experienced an earlier relapse of pelvic pain and sooner surgery than the placebo group.

Bazedoxifene (BZA) is a novel SERM used for the treatment of osteoporosis [162] and antagonizes estrogen-induced uterine endometrial stimulation [21]. In a rat model, BZA reduces the size of endometriotic lesions and decreases proliferating cell nuclear antigen and estrogen receptor expression in the endometrium [273]. A tissue-selective estrogen complex (TSEC) containing BZA and conjugated estrogens (CE) also decreased endometriotic lesion size in a mouse model. The addition of estrogens to BZA did not induce endometrial growth or endometrial hyperplasia and did not reduce the efficacy of the SERM [223]. Therefore, TSEC is a potential novel therapy for endometriosis that could have a high level of efficacy without the side effects of currently available treatments.

SR-16234 is another experimental SERM with antagonistic activity on ERα and partial agonistic activity on ERβ. SR has a regressive effect on the development of murine endometriosis-like lesions, by acting on cell proliferation, angiogenesis, inflammation, and NF-κB phosphorylation A recent trial that investigated this drug in a small group of women with endometriosis and adenomyosis showed that SR-16234 was able to decrease the intensity of pelvic pain and dysmenorrhea [274].

#### Aromatase inhibitors

Aromatase is expressed by endometriotic lesions and in the eutopic endometrium of women with endometriosis causing a local secretion of estrogens, which promote the growth and invasion of endometriotic lesions and favour the onset of pain and prostaglandin-mediated inflammation (Fig. 4) [275].

Aromatase inhibitors (AIs) block estrogen synthesis both in the periphery and in the ovaries [276]. Some clinical studies have shown that third- generation nonsteroidal AIs, such as letrozole and anastrozole, effectively reduced the severity of endometriosis-related pain symptoms; however, their use is limited by several adverse events, such as bone and joint pain, muscle aches, and fatigue [277].

The ESHRE guidelines only recommend the use of AIs in association with COCs or progestins or GnRH-a in patients with drug-resistant pain and surgery-resistant recto-vaginal endometriosis [169]. Currently, a randomized, double-blind, parallel-group, multicenter phase IIb trial is evaluating the efficacy and safety of BAY98-7196 (an intravaginal ring with different doses of anastrozole and LNG), in comparison with placebo and LEU (subcutaneous depot) for treating women with symptomatic endometriosis over a 12-week period (NCT02203331).

## Conclusions

Endometriosis is a chronic disease requiring a lifelong management. Based on patient’s symptoms and the desire of pregnancy, an individualized approach aiming to reduce pain, stress, stress-related comorbidities and to improve QoL should be used for an adequate management [1, 21, 25]. Until a few years ago, the suspect of endometriosis represented an indication for surgery, also used to make the diagnosis through the visualization and histology confirmation of endometriotic lesions. Research development has shown a clear endocrine pathogenesis for endometriosis and thus hormonal therapies represent now a cornerstone of its management, as first choice, before surgery and after surgery in order to reduce the risk of recurrence. The goal is to limit non-indicated surgical procedures because of disease recurrence risk, surgical complications [22, 23, 278], and negative effects on ovarian reserve [279]. The modern approach for endometriosis requires a life-long management plan with the aim of maximizing the use of medical treatment, that can be safely prescribed without histological confirmation of the disease [182, 280–282], and avoiding repeated surgical procedures [20]. Medical hormonal treatment should be the first-line therapeutic option also for patients who have not an immediate desire to become pregnant. Currently, hormonal treatments are the most effective drugs for the treatment of endometriosis and are based on the pathogenic mechanisms involved in the disease. The block of menstruation through an inhibition of HPO axis and consequent amenorrhea or pseudodecidualisation impairs the development or the activity of endometriotic implants. A modern endometriosis management includes a patient-focused approach taking care of overall wellbeing, considering stress, QoL and systemic comorbidities.

## Abbreviations

AIs: Aromatase inhibitors
AMH: Anti-Müllerian hormone
ART: Assisted reproductive technologies
BDNF: Brain-derived neurotrophic factor
BMD: Bone Mass Density
BZA: Bazedoxifene
CE: Conjugated estrogens
COCs: Combined oral contraceptives
COX2: Cyclooxygenase 2
CRH: Corticotropin-releasing hormone
CRHR: Corticotropin-releasing hormone receptor
DIE: Deep infiltrating endometriosis
DNG: Dienogest
DSG: Desogestrel
E2: Estradiol
ENG-: Implant Etonogestrel-releasing subdermal implant
ESHRE: European Society of Human Reproduction and Embryology
FDA: Food and Drug Administration
FSH: Follicle-stimulating hormone
Gn-RH: Gonadotropin-releasing hormone
HDL: High-density lipoprotein
HPA: Hypothalamus–pituitary–adrenal axis
HPO: Hypothalamus-pituitary-ovary axis
IL: Interleukin
IVF: *In vitro* fertilization
LDL: Low-density lipoprotein
LH: Luteinizing hormone
LNG: Levonorgestrel
LNG-IUS: Levonorgestrel intrauterine system
MPA: Medroxyprogesterone acetate
NETA: Norethisterone Acetate
NF-kB: Nuclear factor kappa light-chain-enhancer of activated B cells
NGF: Nerve growth factor
OMA: Ovarian endometriomas
P4: Progesterone
PAECs: Progesterone receptor modulator-associated endometrial changes
PR: Progesterone receptor
QoL: Quality of life
RLX: Raloxifene
ROS: Reactive oxygen species
SERMs: Selective estrogen receptor modulators
SF-1: Steroidogenic factor-1
SNPs: Single nucleotide polymorphisms
SPRMs: Selective progesterone receptor modulators
SRCs: Steroid receptor coactivators
STAR: Steroidogenic acute regulatory protein
SUP: Superficial peritoneal endometriosis
TGF: Transforming growth factors
TNFα: Tumor necrosis factor alpha
TPO: Thyroperoxidase
TSEC: Tissue-selective estrogen complex
TSH: Thyroid stimulating hormone
TSHR: Thyroid stimulating hormone receptor
UCN: Urocortin
UPA: Ulipristal acetate
17β-HSD-2: 17β-Hydroxysteroid dehydrogenase type 2

## References

[1] Chapron C, Marcellin L, Borghese B, Santulli P (2019). Rethinking mechanisms, diagnosis and management of endometriosis. Nat Rev Endocrinol.

[2] Buck Louis GM, Hediger ML, Peterson CM (2011). Incidence of endometriosis by study population and diagnostic method: The ENDO study. Fertil Steril.

[3] Zondervan KT, Becker CM, Koga K, Missmer SA, Taylor RN, Viganò P (2018). Endometriosis Nat Rev Dis Prim.

[4] Reis FM, Petraglia F, Taylor RN (2013). Endometriosis: Hormone regulation and clinical consequences of chemotaxis and apoptosis. Hum Reprod Update.

[5] Reis FM, Coutinho LM, Vannuccini S, Batteux F, Chapron C, Petraglia F (2020). Progesterone receptor ligands for the treatment of endometriosis: The mechanisms behind therapeutic success and failure. Hum Reprod Update.

[6] Patel BG, Lenk EE, Lebovic DI, Shu Y, Yu J, Taylor RN (2018). Pathogenesis of endometriosis: Interaction between Endocrine and inflammatory pathways. Best Pract Res Clin Obstet Gynaecol.

[7] Han SJ, O’Malley BW (2014). The dynamics of nuclear receptors and nuclear receptor coregulators in the pathogenesis of endometriosis. Hum Reprod Update.

[8] Burney RO, Giudice LC (2012). Pathogenesis and pathophysiology of endometriosis. Fertil Steril.

[9] Benagiano G, Brosens I, Habiba M (2014). Structural and molecular features of the endomyometrium in endometriosis and adenomyosis. Hum Reprod Update.

[10] Leyendecker G, Bilgicyildirim A, Inacker M, et al. Adenomyosis and endometriosis. Re-visiting their association and further insights into the mechanisms of auto-traumatisation. An MRI study. Arch Gynecol Obstet. 2015;291(4):917–32. 10.1007/s00404-014-3437-8. 10.1007/s00404-014-3437-8 PMC4355446 25241270

[11] Filippi I, Carrarelli P, Luisi S (2016). Different Expression of Hypoxic and Angiogenic Factors in Human Endometriotic Lesions. Reprod Sci.

[12] Gori M, Luddi A, Belmonte G (2016). Expression of microtubule associated protein 2 and synaptophysin in endometrium: high levels in deep infiltrating endometriosis lesions. Fertil Steril.

[13] Guo SW (2018). Fibrogenesis resulting from cyclic bleeding: The Holy Grail of the natural history of ectopic endometrium. Hum Reprod.

[14] Chamié LP, Ribeiro DMFR, Tiferes DA, De Macedo Neto AC, Serafini PC (2018). Atypical sites of deeply infiltrative endometriosis: Clinical characteristics and imaging findings. Radiographics.

[15] Vannuccini S, Tosti C, Carmona F (2017). Pathogenesis of adenomyosis: An update on molecular mechanisms. Reprod Biomed Online.

[16] Vannuccini S, Petraglia F. Recent advances in understanding and managing adenomyosis. F1000Research. 2019;8. 10.12688/f1000research.17242.1. 10.12688/f1000research.17242.1 PMC6419978 30918629

[17] Agarwal SK, Chapron C, Giudice LC (2019). Clinical diagnosis of endometriosis: a call to action. Am J Obstet Gynecol.

[18] De Graaff AA, D’hooghe TM, Dunselman GAJ, et al. The significant effect of endometriosis on physical, mental and social wellbeing: Results from an international cross-sectional survey. Hum Reprod. 2013;28(10):2677–85. 10.1093/humrep/det284. 10.1093/humrep/det284 23847114

[19] Marinho MCP, Magalhaes TF, Fernandes LFC, Augusto KL, Brilhante AVM, Bezerra LRPS (2018). Quality of Life in Women with Endometriosis: An Integrative Review. J Women’s Heal.

[20] Treatment of pelvic pain associated with endometriosis (2014). A committee opinion. Fertil Steril.

[21] Clemenza S, Sorbi F, Noci I (2018). From pathogenesis to clinical practice: Emerging medical treatments for endometriosis. Best Pract Res Clin Obstet Gynaecol.

[22] Ceccaroni M, Bounous VE, Clarizia R, Mautone D, Mabrouk M (2019). Recurrent endometriosis: a battle against an unknown enemy. Eur J Contracept Reprod Heal Care.

[23] Ianieri MM, Mautone D, Ceccaroni M (2018). Recurrence in Deep Infiltrating Endometriosis: A Systematic Review of the Literature. J Minim Invasive Gynecol.

[24] Ferrero S, Barra F, Leone Roberti Maggiore U. Current and Emerging Therapeutics for the Management of Endometriosis. Drugs. 2018;78(10):995–1012. 10.1007/s40265-018-0928-0. 10.1007/s40265-018-0928-0 29946962

[25] Capezzuoli T, Vannuccini S, Mautone D (2020). Long-term hormonal treatment reduces repetitive surgery for endometriosis recurrence. Reprod Biomed Online.

[26] Barra F, Scala C, Ferrero S (2018). Current understanding on pharmacokinetics, clinical efficacy and safety of progestins for treating pain associated to endometriosis. Expert Opin Drug Metab Toxicol.

[27] Méar L, Herr M, Fauconnier A, Pineau C, Vialard F (2020). Polymorphisms and endometriosis: A systematic review and meta-analyses. Hum Reprod Update.

[28] André GM, Martins Trevisan C, Pedruzzi IN (2018). The impact of FSHR gene polymorphisms Ala307Thr and Asn680Ser in the endometriosis development. DNA Cell Biol.

[29] Wang HS, Cheng BH, Wu HM (2011). A mutant single nucleotide polymorphism of follicle-stimulating hormone receptor is associated with a lower risk of endometriosis. Fertil Steril.

[30] Kerimoglu OS, Yılmaz SA, Pekin A (2015). Follicle-stimulating hormone receptor gene polymorphisms in women with endometriosis. Arch Gynecol Obstet.

[31] Bosco L, Ruvolo G, Luparello C (2017). Gene Expression and Apoptosis Levels in Cumulus Cells of Patients with Polymorphisms of FSHR and LHB Undergoing *in Vitro* Fertilization Program. Cell Physiol Biochem.

[32] Schmitz CR, de Souza CAB, Genro VK, Matte U, de Conto E, Cunha-Filho JS (2015). LH (Trp8Arg/Ile15Thr), LHR (insLQ) and FSHR (Asn680Ser) polymorphisms genotypic prevalence in women with endometriosis and infertility. J Assist Reprod Genet.

[33] Bulun SE, Yilmaz BD, Sison C (2019). Endometriosis. Endocr Rev.

[34] Noble LS, Simpson ER, Johns A, Bulun SE (1996). Aromatase expression in endometriosis. J Clin Endocrinol Metab.

[35] Zeitoun K, Takayama K, Sasano H (1998). Deficient 17β-hydroxysteroid dehydrogenase type 2 expression in endometriosis: Failure to metabolize 17β-estradiol. J Clin Endocrinol Metab.

[36] Bulun SE, Cheng YH, Yin P, et al. Progesterone resistance in endometriosis: Link to failure to metabolize estradiol. Mol Cell Endocrinol. 2006. 10.1016/j.mce.2005.11.041. 10.1016/j.mce.2005.11.041 16406281

[37] Bulun SE, Utsunomiya H, Lin Z (2009). Steroidogenic factor-1 and endometriosis. Mol Cell Endocrinol.

[38] Attar E, Tokunaga H, Imir G (2009). Prostaglandin E2 via steroidogenic factor-1 coordinately regulates transcription of steroidogenic genes necessary for estrogen synthesis in endometriosis. J Clin Endocrinol Metab.

[39] Yilmaz BD, Bulun SE (2019). Endometriosis and nuclear receptors. Hum Reprod Update.

[40] Bulun SE, Monsavais D, Pavone ME (2012). Role of estrogen receptor-β in endometriosis. Semin Reprod Med.

[41] Xue Q, Lin Z, Cheng YH (2007). Promoter methylation regulates estrogen receptor 2 in human endometrium and endometriosis. Biol Reprod.

[42] Patel B, Elguero S, Thakore S, Dahoud W, Bedaiwy M, Mesiano S (2015). Role of nuclear progesterone receptor isoforms in uterine pathophysiology. Hum Reprod Update.

[43] Juhasz-Böss I, Fischer C, Lattrich C (2011). Endometrial expression of estrogen receptor β and its splice variants in patients with and without endometriosis. Arch Gynecol Obstet.

[44] Kumagami A, Ito A, Yoshida-Komiya H, Fujimori K, Sato A (2011). Expression patterns of the steroid receptor coactivator family in human ovarian endometriosis. J Obstet Gynaecol Res.

[45] Han SJ, Hawkins SM, Begum K (2012). A new isoform of steroid receptor coactivator-1 is crucial for pathogenic progression of endometriosis. Nat Med.

[46] Han SJ, Jung SY, Wu SP (2015). Estrogen Receptor β Modulates Apoptosis Complexes and the Inflammasome to Drive the Pathogenesis of Endometriosis. Cell.

[47] Bulun SE, Monsavais D, Pavone ME (2012). Role of estrogen receptor-beta in endometriosis. Semin Reprod Med.

[48] Reis FM, Petraglia F, Taylor RN (2013). Endometriosis: Hormone regulation and clinical consequences of chemotaxis and apoptosis. Hum Reprod Update.

[49] Dmowski WP, Ding J, Shen J, Rana N, Fernandez BB, Braun DP (2001). Apoptosis in endometrial glandular and stromal cells in women with and without endometriosis. Hum Reprod.

[50] Han SJ, Jung SY, Wu SP (2015). Estrogen Receptor β Modulates Apoptosis Complexes and the Inflammasome to Drive the Pathogenesis of Endometriosis. Cell.

[51] Greaves E, Temp J, Esnal-Zufiurre A, Mechsner S, Horne AW, Saunders PTK (2015). Estradiol Is a Critical Mediator of Macrophage-Nerve Cross Talk in Peritoneal Endometriosis. Am J Pathol.

[52] Montagna P, Capellino S, Villaggio B (2008). Peritoneal fluid macrophages in endometriosis: correlation between the expression of estrogen receptors and inflammation. Fertil Steril.

[53] Patel BG, Rudnicki M, Yu J, Shu Y, Taylor RN (2017). Progesterone resistance in endometriosis: origins, consequences and interventions. Acta Obstet Gynecol Scand.

[54] Burney RO, Talbi S, Hamilton AE (2007). Gene expression analysis of endometrium reveals progesterone resistance and candidate susceptibility genes in women with endometriosis. Endocrinology.

[55] Kao LC, Germeyer A, Tulac S (2003). Expression profiling of endometrium from women with endometriosis reveals candidate genes for disease. Based implantation failure and infertility. Endocrinology.

[56] Burney RO, Giudice LC (2012). Pathogenesis and pathophysiology of endometriosis. Fertil Steril.

[57] Attia GR, Zeitoun K, Edwards D, Johns A, Carr BR, Bulun SE (2000). Progesterone receptor isoform A but not B is expressed in endometriosis. J Clin Endocrinol Metab.

[58] Near AM, Wu AH, Templeman C (2011). Progesterone receptor gene polymorphisms and risk of endometriosis: Results from an international collaborative effort. Fertil Steril.

[59] Wieser F, Schneeberger C, Tong D, Tempfer C, Huber JC, Wenzl R (2002). PROGINS receptor gene polymorphism is associated with endometriosis. Fertil Steril.

[60] Pabalan N, Salvador A, Jarjanazi H, Christofolini DM, Barbosa CP, Bianco B (2014). Association of the progesterone receptor gene polymorphism (PROGINS) with endometriosis: a meta-analysis. Arch Gynecol Obstet.

[61] Barragan F, Irwin JC, Balayan S (2016). Human Endometrial Fibroblasts Derived from Mesenchymal Progenitors Inherit Progesterone Resistance and Acquire an Inflammatory Phenotype in the Endometrial Niche in Endometriosis. Biol Reprod.

[62] Florio P, Luisi S, Viganò P (1998). Healthy women and patients with endometriosis show high concentrations of inhibin A, inhibin B, and activin A in peritoneal fluid throughout the menstrual cycle. Hum Reprod.

[63] Reis FM, Di Blasio AM, Florio P, Ambrosini G, Di Loreto C, Petraglia F (2001). Evidence for local production of inhibin A and activin A in patients with ovarian endometriosis. Fertil Steril.

[64] Mabuchi Y, Yamamoto M, Minami S, Umesaki N (2010). Immunohistochemical localization of inhibin and activin subunits, activin receptors and Smads in ovarian endometriosis. Int J Mol Med.

[65] Rocha ALL, Carrarelli P, Novembri R (2012). Activin A stimulates interleukin 8 and vascular endothelial growth factor release from cultured human endometrial stromal cells: possible implications for the pathogenesis of endometriosis. Reprod Sci.

[66] Akiyama I, Yoshino O, Osuga Y (2013). Follistatin is induced by IL-1β and TNF-α in stromal cells from endometrioma. Reprod Sci.

[67] Rocha ALL, Carrarelli P, Novembri R (2011). Altered expression of activin, cripto, and follistatin in the endometrium of women with endometrioma. Fertil Steril.

[68] Cruz C Dela, Del Puerto HL, Rocha ALL, et al. Expression of nodal, cripto, SMAD3, phosphorylated SMAD3, and SMAD4 in the proliferative endometrium of women with endometriosis. Reprod Sci. 2015. 10.1177/1933719114549855. 10.1177/1933719114549855 PMC4519761 25228630

[69] Reis FM, Luisi S, Abro MS (2012). Diagnostic value of serum activin A and follistatin levels in women with peritoneal, ovarian and deep infiltrating endometriosis. Hum Reprod.

[70] Lemos NA, Arbo E, Scalco R, Weiler E, Rosa V, Cunha-Filho JS (2008). Decreased anti-Müllerian hormone and altered ovarian follicular cohort in infertile patients with mild/minimal endometriosis. Fertil Steril.

[71] Pacchiarotti A, Frati P, Milazzo GN, Catalano A, Gentile V, Moscarini M (2014). Evaluation of serum anti-Mullerian hormone levels to assess the ovarian reserve in women with severe endometriosis. Eur J Obstet Gynecol Reprod Biol.

[72] Streuli I, De Ziegler D, Gayet V (2012). In women with endometriosis anti-Müllerian hormone levels are decreased only in those with previous endometrioma surgery. Hum Reprod.

[73] Raffi F, Metwally M, Amer S (2012). The impact of excision of ovarian endometrioma on ovarian reserve: A systematic review and meta-analysis. J Clin Endocrinol Metab.

[74] Somigliana E, Berlanda N, Benaglia L, Viganò P, Vercellini P, Fedele L (2012). Surgical excision of endometriomas and ovarian reserve: a systematic review on serum antimüllerian hormone level modifications. Fertil Steril.

[75] Romanski PA, Brady PC, Farland LV, Thomas AM, Hornstein MD (2019). The effect of endometriosis on the antimüllerian hormone level in the infertile population. J Assist Reprod Genet.

[76] Hwu YM, Wu FS, Li SH, Sun FJ, Lin MH, Lee RK (2011). The impact of endometrioma and laparoscopic cystectomy on serum anti-Müllerian hormone levels. Reprod Biol Endocrinol.

[77] Kasapoglu I, Ata B, Uyaniklar O (2018). Endometrioma-related reduction in ovarian reserve (ERROR): a prospective longitudinal study. Fertil Steril.

[78] Maneschi F, Marasá L, Incandela S, Mazzarese M, Zupi E (1993). Ovarian cortex surrounding benign neoplasms: a histologic study. Am J Obstet Gynecol.

[79] The Effect of Surgery for Endometriomas on Fertility: Scientific Impact Paper No. 55. BJOG. 2018;125(6):e19-28. 10.1111/1471-0528.14834. 10.1111/1471-0528.14834 28944556

[80] Amer SAK. The impact of endometrioma and its surgical treatment on ovarian reserve and reproductive performance. Reprod Surg Assist Concept. 2015:43–57. 10.1007/978-1-4471-4953-8_5.

[81] Kitajima M, Matsumoto K, Murakami N (2020). AMH Concentrations in Peritoneal Fluids of Women With and Without Endometriosis. Front Surg.

[82] Wang J, Dicken C, Lustbader JW, Tortoriello DV (2009). Evidence for a Müllerian-inhibiting substance autocrine/paracrine system in adult human endometrium. Fertil Steril.

[83] Namkung J, Song JY, Jo HH (2012). Mullerian inhibiting substance induces apoptosis of human endometrial stromal cells in endometriosis. J Clin Endocrinol Metab.

[84] Signorile PG, Petraglia F, Baldi A (2014). Anti-mullerian hormone is expressed by endometriosis tissues and induces cell cycle arrest and apoptosis in endometriosis cells. J Exp Clin Cancer Res.

[85] Galhardo A, Moura-Ramos M, Cunha M, Pinto-Gouveia J (2016). The infertility trap: How defeat and entrapment affect depressive symptoms. Hum Reprod.

[86] Siedentopf F, Tariverdian N, Rücke M, Kentenich H, Arck PC (2008). Immune status, psychosocial distress and reduced quality of life in infertile patients with endometriosis. Am J Reprod Immunol.

[87] Soliman AM, Coyne KS, Gries KS, Castelli-Haley J, Snabes MC, Surrey ES (2017). The effect of endometriosis symptoms on absenteeism and presenteeism in the workplace and at home. J Manag Care Spec Pharm.

[88] Luisi S, Pizzo A, Pinzauti S, et al. Neuroendocrine and stress-related aspects of endometriosis. Neuroendocrinol Lett. 2015. PMID: 25789593. 25789593

[89] Harrison V, Rowan K, Mathias J (2005). Stress reactivity and family relationships in the development and treatment of endometriosis. Fertil Steril.

[90] Staal AHJ, Van Der Zanden M, Nap AW (2016). Diagnostic Delay of Endometriosis in the Netherlands. Gynecol Obstet Invest.

[91] Soliman AM, Fuldeore M, Snabes MC (2017). Factors Associated with Time to Endometriosis Diagnosis in the United States. J Women’s Heal.

[92] Lazzeri L, Vannuccini S, Orlandini C (2015). Surgical treatment affects perceived stress differently in women with endometriosis: Correlation with severity of pain. Fertil Steril.

[93] Lazzeri L, Orlandini C, Vannuccini S, Pinzauti S, Tosti C, Zupi E, Nappi RE, Petraglia F (2015). Endometriosis and Perceived Stress : Impact of Surgical and Medical Treatment. Gynecol Obstet Invest.

[94] Facchin F, Barbara G, Saita E (2015). Impact of endometriosis on quality of life and mental health: Pelvic pain makes the difference. J Psychosom Obstet Gynecol.

[95] Márki G, Bokor A, Rigó J, Rigó A (2017). Physical pain and emotion regulation as the main predictive factors of health-related quality of life in women living with endometriosis. Hum Reprod.

[96] Facchin F, Barbara G, Dridi D (2017). Mental health in women with endometriosis: Searching for predictors of psychological distress. Hum Reprod.

[97] Coxon L, Horne AW, Vincent K (2018). Pathophysiology of endometriosis-associated pain: A review of pelvic and central nervous system mechanisms. Best Pract Res Clin Obstet Gynaecol.

[98] Frodl T, O’Keane V (2013). How does the brain deal with cumulative stress? A review with focus on developmental stress, HPA axis function and hippocampal structure in humans. Neurobiol Dis.

[99] Fries E, Hesse J, Hellhammer J, Hellhammer DH (2005). A new view on hypocortisolism. Psychoneuroendocrinology.

[100] Blackburn-Munro G, Blackburn-Munro R (2003). Pain in the brain: Are hormones to blame?. Trends Endocrinol Metab.

[101] Ortiz R, Gemmill JAL, Sinaii N (2020). Hypothalamic-Pituitary-Adrenal Axis Responses in Women with Endometriosis-Related Chronic Pelvic Pain. Reprod Sci.

[102] Petrelluzzi KFS, Garcia MC, Petta CA, Grassi-Kassisse DM, Spadari-Bratfisch RC (2008). Salivary cortisol concentrations, stress and quality of life in women with endometriosis and chronic pelvic pain. Stress.

[103] Quiñones M, Urrutia R, Torres-Reverón A, Vincent K, Flores I (2015). Anxiety, coping skills and hypothalamus-pituitary-adrenal (HPA) axis in patients with endometriosis. J Reprod Biol Heal.

[104] van Aken M, Oosterman J, van Rijn T (2018). Hair cortisol and the relationship with chronic pain and quality of life in endometriosis patients. Psychoneuroendocrinology.

[105] Lima AP, Moura MD, Rosa e Silva AAM. Prolactin and cortisol levels in women with endometriosis. Brazilian J Med Biol Res. 2006. 10.1590/S0100-879X2006000800015. 10.1590/s0100-879x2006000800015 16906287

[106] Friggi Sebe Petrelluzzi K, Garcia MC, Petta CA, et al. Physical therapy and psychological intervention normalize cortisol levels and improve vitality in women with endometriosis. J Psychosom Obstet Gynecol. 2012. 10.3109/0167482X.2012.729625. 10.3109/0167482X.2012.729625 23094607

[107] Petraglia F, Imperatore A, Challis JRG (2010). Neuroendocrine mechanisms in pregnancy and parturition. Endocr Rev.

[108] Novembri R, Borges LE, Carrarelli P (2011). Impaired CRH and urocortin expression and function in eutopic endometrium of women with endometriosis. J Clin Endocrinol Metab.

[109] Carrarelli P, Luddi A, Funghi L (2016). Urocortin and corticotrophin-releasing hormone receptor type 2 mRNA are highly expressed in deep infiltrating endometriotic lesions. Reprod Biomed Online.

[110] Novembri R, Carrarelli P, Toti P (2011). Urocortin 2 and urocortin 3 in endometriosis: evidence for a possible role in inflammatory response. Mol Hum Reprod.

[111] Florio P, Reis F, Torres P (2007). Plasma urocortin levels in the diagnosis of ovarian endometriosis. Obstet Gynecol.

[112] Maia LM, Rocha AL, Del Puerto HL, Petraglia F, Reis FM (2018). Plasma urocortin-1 as a preoperative marker of endometriosis in symptomatic women. Gynecol Endocrinol.

[113] Sinaii N, Cleary SD, Ballweg ML, Nieman LK, Stratton P (2002). High rates of autoimmune and endocrine disorders, fibromyalgia, chronic fatigue syndrome and atopic diseases among women with endometriosis: a survey analysis. Hum Reprod.

[114] Poppe K, Velkeniers B, Glinoer D (2008). The role of thyroid autoimmunity in fertility and pregnancy. Nat Clin Pract Endocrinol Metab.

[115] Yuk JS, Park EJ, Seo YS, Kim HJ, Kwon SY, Park WI (2016). Graves disease is associated with endometriosis: A 3-year population-based cross-sectional study. Med (United States).

[116] Aghajanova L, Giudice LC (2011). Molecular evidence for differences in endometrium in severe versus mild endometriosis. Reprod Sci.

[117] Peyneau M, Kavian N, Chouzenoux S (2019). Role of thyroid dysimmunity and thyroid hormones in endometriosis. Proc Natl Acad Sci U S A.

[118] Mousa SA, O’Connor LJ, Bergh JJ, Davis FB, Scanlan TS, Davis PJ (2005). The proangiogenic action of thyroid hormone analogue GC-1 is initiated at an integrin. J Cardiovasc Pharmacol.

[119] Stavreus EA (2012). Paracrine interactions of thyroid hormones and thyroid stimulation hormone in the female reproductive tract have an impact on female fertility. Front Endocrinol (Lausanne).

[120] DiVasta AD, Vitonis AF, Laufer MR, Missmer SA (2018). Spectrum of symptoms in women diagnosed with endometriosis during adolescence vs adulthood. Am J Obstet Gynecol.

[121] Fauconnier A, Chapron C (2005). Endometriosis and pelvic pain: Epidemiological evidence of the relationship and implications. Hum Reprod Update.

[122] Schliep KC, Mumford SL, Peterson CM (2015). Pain typology and incident endometriosis. Hum Reprod.

[123] Ballard K, Lane H, Hudelist G, Banerjee S, Wright J (2010). Can specific pain symptoms help in the diagnosis of endometriosis? A cohort study of women with chronic pelvic pain. Fertil Steril.

[124] Chung MK, Chung RP, Gordon D. Interstitial cystitis and endometriosis in patients with chronic pelvic pain: The “Evil Twins” syndrome. JSLS. 2005. PMC3015562 15791965

[125] Redwine DB (2002). Diaphragmatic endometriosis: diagnosis, surgical management, and long-term results of treatment. Fertil Steril.

[126] Fedele L, Berlanda N, Corsi C, Gazzano G, Morini M, Vercellini P (2014). Ileocecal endometriosis: Clinical and pathogenetic implications of an underdiagnosed condition. Fertil Steril.

[127] Coratti F, Vannuccini S, Foppa C (2020). Emergency surgery for appendectomy and incidental diagnosis of superficial peritoneal endometriosis in fertile age women. Reprod Biomed Online.

[128] Morotti M, Vincent K, Becker CM (2017). Mechanisms of pain in endometriosis. Eur J Obstet Gynecol Reprod Biol.

[129] Stratton P, Berkley KJ (2011). Chronic pelvic pain and endometriosis: Translational evidence of the relationship and implications. Hum Reprod Update.

[130] Morotti M, Vincent K, Brawn J, Zondervan KT, Becker CM (2014). Peripheral changes in endometriosis-associated pain. Hum Reprod Update.

[131] Brawn J, Morotti M, Zondervan KT, Becker CM, Vincent K (2014). Central changes associated with chronic pelvic pain and endometriosis. Hum Reprod Update.

[132] Barcena de Arellano ML, Arnold J, Lang H, et al. Evidence of neurotrophic events due to peritoneal endometriotic lesions. Cytokine. 2013. 10.1016/j.cyto.2013.03.003. 10.1016/j.cyto.2013.03.003 23545214

[133] Krizsan-Agbas D, Pedchenko T, Hasan W, Smith PG (2003). Oestrogen regulates sympathetic neurite outgrowth by modulating brain derived neurotrophic factor synthesis and release by the rodent uterus. Eur J Neurosci.

[134] Tokushige N, Markham R, Russell P, Fraser IS (2006). Nerve fibres in peritoneal endometriosis. Hum Reprod.

[135] Wang G, Tokushige N, Markham R, Fraser IS (2009). Rich innervation of deep infiltrating endometriosis. Hum Reprod.

[136] Riccio L da GC, Santulli P, Marcellin L, Abrão MS, Batteux F, Chapron C. Immunology of endometriosis. Best Pract Res Clin Obstet Gynaecol. 2018. 10.1016/j.bpobgyn.2018.01.010. 10.1016/j.bpobgyn.2018.01.010 29506962

[137] Woolf CJ (2011). Central sensitization: Implications for the diagnosis and treatment of pain. Pain.

[138] As-Sanie S, Harris RE, Napadow V (2012). Changes in regional gray matter volume in women with chronic pelvic pain: A voxel-based morphometry study. Pain.

[139] As-Sanie S, Kim J, Schmidt-Wilcke T, et al. Functional Connectivity Is Associated with Altered Brain Chemistry in Women with Endometriosis-Associated Chronic Pelvic Pain. J Pain. 2016. 10.1016/j.jpain.2015.09.008. 10.1016/j.jpain.2015.09.008 PMC4698023 26456676

[140] Vannuccini S, Lazzeri L, Orlandini C (2017). Mental health, pain symptoms and systemic comorbidities in women with endometriosis: a cross-sectional study. J Psychosom Obstet Gynecol.

[141] McPeak AE, Allaire C, Williams C, Albert A, Lisonkova S, Yong PJ. Pain Catastrophizing and pain health-related quality-of-life in endometriosis. Clin J Pain. 2018;34. 10.1097/AJP.0000000000000539 28731958

[142] Laganà AS, La Rosa VL, Rapisarda AMC (2017). Anxiety and depression in patients with endometriosis: Impact and management challenges. Int J Womens Health.

[143] Álvarez-Salvago F, Lara-Ramos A, Cantarero-Villanueva I (2020). Chronic fatigue, physical impairments and quality of life in women with endometriosis: A case-control study. Int J Environ Res Public Health.

[144] Jones GT. Psychosocial vulnerability and early life adversity as risk factors for central sensitivity syndromes. Curr Rheumatol Rev. 2016. 10.2174/1573397112666151231113438. 10.2174/1573397112666151231113438 26717947

[145] Kvaskoff M, Mu F, Terry KL (2015). Endometriosis: a high-risk population for major chronic diseases?. Hum Reprod Update.

[146] Jess T, Frisch M, Jorgensen KT, Pedersen BV, Nielsen NM (2012). Increased risk of inflammatory bowel disease in women with endometriosis: a nationwide Danish cohort study. Gut.

[147] Bungum HF, Vestergaard C, Knudsen UB (2014). Endometriosis and type 1 allergies/immediate type hypersensitivity: a systematic review. Eur J Obstet Gynecol Reprod Biol.

[148] Caserta D, Mallozzi M, Pulcinelli FM, Mossa B, Moscarini M. Endometriosis allergic or autoimmune disease: pathogenetic aspects--a case control study. Clin Exp Obs Gynecol. 2016;43. PMID: 27328490. 27328490

[149] Harris HR, Costenbader KH, Mu F, et al. Endometriosis and the risks of systemic lupus erythematosus and rheumatoid arthritis in the Nurses’ health study II. Ann Rheum Dis. 2016;75. 10.1136/annrheumdis-2015-207704. 10.1136/annrheumdis-2015-207704 PMC4740245 26238146

[150] Greenbaum H, Weil C, Chodick G, Shalev V, Eisenberg VH. Evidence for an association between endometriosis, fibromyalgia, and autoimmune diseases. Am J Reprod Immunol. 2019;81. 10.1111/aji.13095. 10.1111/aji.13095 30682223

[151] Nielsen NM, Jorgensen KT, Pedersen B V, Rostgaard K, Frisch M. The co-occurrence of endometriosis with multiple sclerosis, systemic lupus erythematosus and Sjogren syndrome. Hum Reprod. 2011;26. 10.1093/humrep/der105. 10.1093/humrep/der105 21471158

[152] Tariverdian N, Theoharides TC, Siedentopf F, et al. Neuroendocrine-immune disequilibrium and endometriosis: an interdisciplinary approach. Semin Immunopathol. 2007;29. 10.1007/s00281-007-0077-0. 10.1007/s00281-007-0077-0 PMC2668599 17621704

[153] Shigesi N, Kvaskoff M, Kirtley S (2019). The association between endometriosis and autoimmune diseases: A systematic review and meta-analysis. Hum Reprod Update.

[154] Tomassetti C, D’Hooghe T (2018). Endometriosis and infertility: Insights into the causal link and management strategies. Best Pract Res Clin Obstet Gynaecol.

[155] Tanbo T, Fedorcsak P (2017). Endometriosis-associated infertility: aspects of pathophysiological mechanisms and treatment options. Acta Obstet Gynecol Scand.

[156] Dueholm M (2017). Uterine adenomyosis and infertility, review of reproductive outcome after *in vitro* fertilization and surgery. Acta Obstet Gynecol Scand.

[157] Campo S, Campo V, Benagiano G (2012). Adenomyosis and infertility. Reprod Biomed Online.

[158] Lessey BA, Kim JJ (2017). Endometrial receptivity in the eutopic endometrium of women with endometriosis: it is affected, and let me show you why. Fertil Steril.

[159] Miravet-Valenciano J, Ruiz-Alonso M, Gómez E, Garcia-Velasco JA (2017). Endometrial receptivity in eutopic endometrium in patients with endometriosis: it is not affected, and let me show you why. Fertil Steril.

[160] Barra F, Grandi G, Tantari M, Scala C, Facchinetti F, Ferrero S (2019). A comprehensive review of hormonal and biological therapies for endometriosis: latest developments. Expert Opin Biol Ther.

[161] Levine D, Kaufman L, Cuenca VG, Badawy SZA (2007). Cell growth effects of leuprolide on cultured endometrioma cells. J Reprod Med.

[162] Barra F, Grandi G, Tantari M, Scala C, Facchinetti F, Ferrero S (2019). A comprehensive review of hormonal and biological therapies for endometriosis: latest developments. Expert Opin Biol Ther.

[163] Fedele L, Bianchi S, Bocciolone L, Di Nola G, Franchi D (1993). Buserelin acetate in the treatment of pelvic pain associated with minimal and mild endometriosis: a controlled study. Fertil Steril.

[164] Bergqvist A, Bergh T, Hogström L, Mattsson S, Nordenskjöld F, Rasmussen C (1998). Effects of triptorelin versus placebo on the symptoms of endometriosis. Fertil Steril.

[165] Dlugi AM, Miller JD, Knittle J (1990). Lupron depot (leuprolide acetate for depot suspension) in the treatment of endometriosis: a randomized, placebo-controlled, double-blind study. Lupron Study Group Fertil Steril.

[166] Miller JD (1990). Leuprolide acetate for the treatment of endometriosis. Prog Clin Biol Res.

[167] Brown J, Pan A, Hart RJ. Gonadotrophin-releasing hormone analogues for pain associated with endometriosis. Cochrane database Syst Rev. 2010. 10.1002/14651858.CD008475.pub2. 10.1002/14651858.CD008475.pub2 PMC7388859 21154398

[168] Sallam HN, Garcia-Velasco JA, Dias S, Arici A. Long-term pituitary down-regulation before *in vitro* fertilization (IVF) for women with endometriosis. Cochrane database Syst Rev. 2006. 10.1002/14651858.CD004635.pub2. 10.1002/14651858.CD004635.pub2 PMC8195082 16437491

[169] Dunselman GAJ, Vermeulen N, Becker C (2014). ESHRE guideline: management of women with endometriosis. Hum Reprod.

[170] Tosti C, Biscione A, Morgante G, Bifulco G, Luisi S, Petraglia F (2016). Ac ce pt cr t. Eur J Obstet Gynecol.

[171] Mitwally MFM, Gotlieb L, Casper RF (2002). Prevention of bone loss and hypoestrogenic symptoms by estrogen and interrupted progestogen add-back in long-term GnRH-agonist down-regulated patients with endometriosis and premenstrual syndrome. Menopause.

[172] Bedaiwy MA, Casper RF (2006). Treatment with leuprolide acetate and hormonal add-back for up to 10 years in stage IV endometriosis patients with chronic pelvic pain. Fertil Steril.

[173] Taylor HS, Giudice LC, Lessey BA (2017). Treatment of endometriosis-associated pain with elagolix, an oral GnRH antagonist. N Engl J Med.

[174] Agarwal SK, Singh SS, Archer DF (2021). Endometriosis-Related Pain Reduction During Bleeding and Nonbleeding Days in Women Treated with Elagolix. J Pain Res.

[175] Surrey E, Taylor HS, Giudice L (2018). Long-term outcomes of elagolix in women with endometriosis results from two extension studies. Obstet Gynecol.

[176] Pokrzywinski RM, Soliman AM, Chen J (2020). Achieving clinically meaningful response in endometriosis pain symptoms is associated with improvements in health-related quality of life and work productivity: analysis of 2 phase III clinical trials. Am J Obstet Gynecol.

[177] Surrey ES, Soliman AM, Agarwal SK, Snabes MC, Diamond MP (2019). Impact of elagolix treatment on fatigue experienced by women with moderate to severe pain associated with endometriosis. Fertil Steril.

[178] Ng J, Chwalisz K, Carter DC, Klein CE (2017). Dose-dependent suppression of gonadotropins and ovarian hormones by elagolix in healthy premenopausal women. J Clin Endocrinol Metab.

[179] Struthers RS, Nicholls AJ, Grundy J (2009). Suppression of gonadotropins and estradiol in premenopausal women by oral administration of the nonpeptide gonadotropin-releasing hormone antagonist elagolix. J Clin Endocrinol Metab.

[180] Osuga Y, Seki Y, Tanimoto M, Kusumoto T, Kudou K, Terakawa N. Relugolix, an oral gonadotropin-releasing hormone receptor antagonist, reduces endometriosis-associated pain in a dose–response manner: a randomized, double-blind, placebo-controlled study. Fertil Steril. 2020:1–8. 10.1016/j.fertnstert.2020.07.055. 10.1016/j.fertnstert.2020.07.055 32912633

[181] Donnez J, Taylor HS, Taylor RN (2020). Treatment of endometriosis-associated pain with linzagolix, an oral gonadotropin-releasing hormone–antagonist: a randomized clinical trial. Fertil Steril.

[182] Dunselman GAJ, Vermeulen N, Becker C (2014). ESHRE guideline: Management of women with endometriosis. Hum Reprod.

[183] Vercellini P, Buggio L, Berlanda N, Barbara G, Somigliana E, Bosari S (2016). Estrogen-progestins and progestins for the management of endometriosis. Fertil Steril.

[184] Quaas AM, Weedin EA, Hansen KR (2015). On-label and off-label drug use in the treatment of endometriosis. Fertil Steril.

[185] Vercellini P, Buggio L, Frattaruolo MP, Borghi A, Dridi D, Somigliana E (2018). Medical treatment of endometriosis-related pain. Best Pract Res Clin Obstet Gynaecol.

[186] Lee JH, Song JY, Yi KW (2018). Effectiveness of Dienogest for Treatment of Recurrent Endometriosis: Multicenter Data. Reprod Sci.

[187] Techatraisak K, Hestiantoro A, Ruey S (2019). Effectiveness of dienogest in improving quality of life in Asian women with endometriosis (ENVISIOeN): interim results from a prospective cohort study under real-life clinical practice. BMC Womens Health.

[188] García Uranga-Romano J, Hernández-Valencia M, Zárate A, Basavilvazo-Rodríguez MA. Dienogest usefulness in pelvic pain due to endometriosis. A meta-analysis of its effectiveness. Rev Med Inst Mex Seguro Soc. 2017;55(4):452–55. PMID: 28591499. 28591499

[189] Momoeda M, Harada T, Terakawa N (2009). Long-term use of dienogest for the treatment of endometriosis. J Obstet Gynaecol Res.

[190] Muzii L, Galati G, Di Tucci C (2020). Medical treatment of ovarian endometriomas: a prospective evaluation of the effect of dienogest on ovarian reserve, cyst diameter, and associated pain. Gynecol Endocrinol Off J Int Soc Gynecol Endocrinol.

[191] Vignali M, Belloni GM, Pietropaolo G (2020). Effect of Dienogest therapy on the size of the endometrioma. Gynecol Endocrinol.

[192] Angioni S, Pontis A, Malune ME (2020). Is dienogest the best medical treatment for ovarian endometriomas? Results of a multicentric case control study. Gynecol Endocrinol.

[193] Del Forno S, Mabrouk M, Arena A (2019). Dienogest or Norethindrone acetate for the treatment of ovarian endometriomas: Can we avoid surgery?. Eur J Obstet Gynecol Reprod Biol.

[194] Papíková Z, Hudeček R, Ventruba P, Szypulová M (2019). Efficacy of dienogest treatment of clinical symptoms of rectovaginal endometriosis. Ces Gynekol.

[195] Leone Roberti Maggiore U, Ferrero S, Candiani M, Somigliana E, Viganò P, Vercellini P. Bladder Endometriosis: A Systematic Review of Pathogenesis, Diagnosis, Treatment, Impact on Fertility, and Risk of Malignant Transformation. Eur Urol. 2017;71(5):790–807. 10.1016/j.eururo.2016.12.015. 10.1016/j.eururo.2016.12.015 28040358

[196] Angioni S, Nappi L, Pontis A, et al. Dienogest. A possible conservative approach in bladder endometriosis. Results of a pilot study. Gynecol Endocrinol Off J Int Soc Gynecol Endocrinol. 2015;31(5):406–8. 10.3109/09513590.2015.1006617. 10.3109/09513590.2015.1006617 25776993

[197] Leonardo-Pinto JP, Benetti-Pinto CL, Cursino K, Yela DA (2017). Dienogest and deep infiltrating endometriosis: The remission of symptoms is not related to endometriosis nodule remission. Eur J Obstet Gynecol Reprod Biol.

[198] Leonardo-Pinto JP, Benetti-Pinto CL, Cursino K, Yela DA (2017). Dienogest and deep infiltrating endometriosis: The remission of symptoms is not related to endometriosis nodule remission. Eur J Obstet Gynecol Reprod Biol.

[199] Caruso S, Iraci M, Cianci S, Vitale SG, Fava V, Cianci A (2019). Effects of long-term treatment with Dienogest on the quality of life and sexual function of women affected by endometriosis-associated pelvic pain. J Pain Res.

[200] Techatraisak K, Hestiantoro A, Ruey S (2019). Effectiveness of dienogest in improving quality of life in Asian women with endometriosis (ENVISIOeN): Interim results from a prospective cohort study under real-life clinical practice. BMC Womens Health.

[201] Yu Q, Zhang S, Li H (2019). Dienogest for treatment of endometriosis in women: A 28-week, open-label, extension study. J Women’s Heal.

[202] Römer T (2018). Long-term treatment of endometriosis with dienogest: retrospective analysis of efficacy and safety in clinical practice. Arch Gynecol Obstet.

[203] Zakhari A, Edwards D, Ryu M, Matelski JJ, Bougie O, Murji A (2020). Dienogest and the Risk of Endometriosis Recurrence Following Surgery: A Systematic Review and Meta-analysis. J Minim Invasive Gynecol.

[204] Cho BS, Roh JW, Park J (2020). Safety and Effectiveness of Dienogest (Visanne®) for Treatment of Endometriosis: A Large Prospective Cohort Study. Reprod Sci.

[205] Lee DY, Lee JY, Seo JW, Yoon BK, Choi DS (2016). Gonadotropin-releasing hormone agonist with add–back treatment is as effective and tolerable as dienogest in preventing pain recurrence after laparoscopic surgery for endometriosis. Arch Gynecol Obstet.

[206] Seo JW, Lee DY, Kim SE, Yoon BK, Choi DS (2019). Comparison of long-term use of combined oral contraceptive after gonadotropin-releasing hormone agonist plus add-back therapy versus dienogest to prevent recurrence of ovarian endometrioma after surgery. Eur J Obstet Gynecol Reprod Biol.

[207] Koshiba A, Mori T, Okimura H (2018). Dienogest therapy during the early stages of recurrence of endometrioma might be an alternative therapeutic option to avoid repeat surgeries. J Obstet Gynaecol Res.

[208] Vercellini P, Bracco B, Mosconi P (2016). Norethindrone acetate or dienogest for the treatment of symptomatic endometriosis: A before and after study. Fertil Steril.

[209] Vercellini P, Pietropaolo G, De Giorgi O, Pasin R, Chiodini A, Crosignani PG (2005). Treatment of symptomatic rectovaginal endometriosis with an estrogen-progestogen combination versus low-dose norethindrone acetate. Fertil Steril.

[210] Ferrero S, Camerini G, Ragni N, Venturini PL, Biscaldi E, Remorgida V (2010). Norethisterone acetate in the treatment of colorectal endometriosis: A pilot study. Hum Reprod.

[211] Morotti M, Venturini PL, Biscaldi E (2017). Efficacy and acceptability of long-term norethindrone acetate for the treatment of rectovaginal endometriosis. Eur J Obstet Gynecol Reprod Biol.

[212] Scala C, Leone Roberti Maggiore U, Barra F, Venturini PL, Ferrero S. Norethindrone acetate versus extended-cycle oral contraceptive (Seasonique ®) in the treatment of endometriosis symptoms: A prospective open-label comparative study. Eur J Obstet Gynecol Reprod Biol. 2018. 10.1016/j.ejogrb.2018.01.022. 10.1016/j.ejogrb.2018.01.022 29408753

[213] Morotti M, Sozzi F, Remorgida V, Venturini PL, Ferrero S (2014). Dienogest in women with persistent endometriosis-related pelvic pain during norethisterone acetate treatment. Eur J Obstet Gynecol Reprod Biol.

[214] Brown J, Kives S, Akhtar M. Progestagens and anti-progestagens for pain associated with endometriosis. Cochrane database Syst Rev. 2012. 10.1002/14651858.CD002122.pub2. 10.1002/14651858.CD002122.pub2 PMC6885053 22419284

[215] Telimaa S, Puolakka J, Rönnberg L, Kauppila A (1987). Placebo-controlled comparison of danazol and high-dose medroxyprogesterone acetate in the treatment of endometriosis. Gynecol Endocrinol Off J Int Soc Gynecol Endocrinol.

[216] Crosignani PG, Luciano A, Ray A, Bergqvist A (2006). Subcutaneous depot medroxyprogesterone acetate versus leuprolide acetate in the treatment of endometriosis-associated pain. Hum Reprod.

[217] Schlaff WD, Carson SA, Luciano A, Ross D, Bergqvist A (2006). Subcutaneous injection of depot medroxyprogesterone acetate compared with leuprolide acetate in the treatment of endometriosis-associated pain. Fertil Steril.

[218] Committee Opinion No (2014). 602: Depot medroxyprogesterone acetate and bone effects. Obstet Gynecol.

[219] Olive DL (2003). Medical therapy of endometriosis. Semin Reprod Med.

[220] Selak V, Farquhar C, Prentice A, Singla A. Danazol for pelvic pain associated with endometriosis. Cochrane database Syst Rev. 2007. 10.1002/14651858.CD000068.pub2. 10.1002/14651858.CD000068.pub2 PMC12740266 17943735

[221] Cobellis L, Razzi S, Fava A, Severi FM, Igarashi M, Petraglia F (2004). A danazol-loaded intrauterine device decreases dysmenorrhea, pelvic pain, and dyspareunia associated with endometriosis. Fertil Steril.

[222] Igarashi M, Iizuka M, Abe Y, Ibuki Y (1998). Novel vaginal danazol ring therapy for pelvic endometriosis, in particular deeply infiltrating endometriosis. Hum Reprod.

[223] Razzi S, Luisi S, Calonaci F, Altomare A, Bocchi C, Petraglia F (2007). Efficacy of vaginal danazol treatment in women with recurrent deeply infiltrating endometriosis. Fertil Steril.

[224] Godin R, Marcoux V. Vaginally Administered Danazol: An Overlooked Option in the Treatment of Rectovaginal Endometriosis? J Obstet Gynaecol Canada JOGC = J d’obstetrique Gynecol du Canada JOGC. 2015;37(12):1098–103. 10.1016/s1701-2163(16)30075-5 10.1016/s1701-2163(16)30075-5 26637082

[225] Okamura Y, Suzuki J, Honda R, Ohba T, Katabuchi H, Okamura H (2008). Clinical outcome of vaginal danazol suppository use in women with pelvic endometriosis. Fertil Steril.

[226] Bhattacharya SM, Tolasaria A, Khan B (2011). Vaginal danazol for the treatment of endometriosis-related pelvic pain. Int J Gynecol Obstet.

[227] Razzi S, Luisi S, Ferretti C (2007). Use of a progestogen only preparation containing desogestrel in the treatment of recurrent pelvic pain after conservative surgery for endometriosis. Eur J Obstet Gynecol Reprod Biol.

[228] Morotti M, Remorgida V, Venturini PL, Ferrero S (2014). Progestin-only contraception compared with extended combined oral contraceptive in women with migraine without aura: a retrospective pilot study. Eur J Obstet Gynecol Reprod Biol.

[229] Leone Roberti Maggiore U, Remorgida V, Scala C, Tafi E, Venturini PL, Ferrero S. Desogestrel-only contraceptive pill versus sequential contraceptive vaginal ring in the treatment of rectovaginal endometriosis infiltrating the rectum: a prospective open-label comparative study. Acta Obstet Gynecol Scand. 2014;93(3):239–47. 10.1111/aogs.12326. 10.1111/aogs.12326 24372517

[230] Viganò P, Somigliana E, Vercellini P (2007). Levonorgestrel-releasing intrauterine system for the treatment of endometriosis: Biological and clinical evidence. Women’s Heal.

[231] Vercellini P, Frontino G, De Giorgi O, Aimi G, Zaina B, Crosignani PG (2003). Comparison of a levonorgestrel-releasing intrauterine device versus expectant management after conservative surgery for symptomatic endometriosis: a pilot study. Fertil Steril.

[232] Bayoglu Tekin Y, Dilbaz B, Altinbas SK, Dilbaz S (2011). Postoperative medical treatment of chronic pelvic pain related to severe endometriosis: levonorgestrel-releasing intrauterine system versus gonadotropin-releasing hormone analogue. Fertil Steril.

[233] Tanmahasamut P, Rattanachaiyanont M, Angsuwathana S, Techatraisak K, Indhavivadhana S, Leerasiri P (2012). Postoperative levonorgestrel-releasing intrauterine system for pelvic endometriosis-related pain: a randomized controlled trial. Obstet Gynecol.

[234] Yucel N, Baskent E, Karamustafaoglu Balci B, Goynumer G (2018). The levonorgestrel-releasing intrauterine system is associated with a reduction in dysmenorrhoea and dyspareunia, a decrease in CA 125 levels, and an increase in quality of life in women with suspected endometriosis. Aust New Zeal J Obstet Gynaecol.

[235] Lee KH, Jung YW, Song SY (2018). Comparison of the efficacy of diegnogest and levonorgestrel-releasing intrauterine system after laparoscopic surgery for endometriosis. J Obstet Gynaecol Res.

[236] Chen Y-J, Hsu T-F, Huang B-S, Tsai H-W, Chang Y-H, Wang P-H (2017). Postoperative maintenance levonorgestrel-releasing intrauterine system and endometrioma recurrence: a randomized controlled study. Am J Obstet Gynecol.

[237] Wattanayingcharoenchai R, Rattanasiri S, Charakorn C, Attia J, Thakkinstian A (2021). Postoperative hormonal treatment for prevention of endometrioma recurrence after ovarian cystectomy: a systematic review and network meta-analysis. BJOG An Int J Obstet Gynaecol.

[238] Song J, Wang Y, Yu L (2018). Clinical comparison of mifepristone and gestrinone for laparoscopic endometriosis. Pak J Pharm Sci.

[239] Walch K, Unfried G, Huber J (2009). Implanon versus medroxyprogesterone acetate: effects on pain scores in patients with symptomatic endometriosis–a pilot study. Contraception.

[240] Yisa SB, Okenwa AA, Husemeyer RP (2005). Treatment of pelvic endometriosis with etonogestrel subdermal implant (Implanon). J Fam Plan Reprod Heal care.

[241] Carvalho N, Margatho D, Cursino K, Benetti-Pinto CL, Bahamondes L (2018). Control of endometriosis-associated pain with etonogestrel-releasing contraceptive implant and 52-mg levonorgestrel-releasing intrauterine system: randomized clinical trial. Fertil Steril.

[242] Alio L, Angioni S, Arena S, et al. When more is not better: 10 ‘don’ts’ in endometriosis management. An ETIC* position statement. Hum Reprod Open. 2019. 10.1093/hropen/hoz009. 10.1093/hropen/hoz009 PMC6560357 31206037

[243] Meresman GF, Augé L, Barañao RI, Lombardi E, Tesone M, Sueldo C (2002). Oral contraceptives suppress cell proliferation and enhance apoptosis of eutopic endometrial tissue from patients with endometriosis. Fertil Steril.

[244] Harada T, Momoeda M, Taketani Y, Hoshiai H, Terakawa N (2008). Low-dose oral contraceptive pill for dysmenorrhea associated with endometriosis: a placebo-controlled, double-blind, randomized trial. Fertil Steril.

[245] Harada T, Kosaka S, Elliesen J, Yasuda M, Ito M, Momoeda M (2017). Ethinylestradiol 20 μg/drospirenone 3 mg in a flexible extended regimen for the management of endometriosis-associated pelvic pain: a randomized controlled trial. Fertil Steril.

[246] Brown J, Crawford TJ, Datta S, Prentice A. Oral contraceptives for pain associated with endometriosis. Cochrane Database Syst Rev. 2018. 10.1002/14651858.CD001019.pub3. 10.1002/14651858.CD001019.pub3 PMC6494634 29786828

[247] Becker CM, Gattrell WT, Gude K, Singh SS (2017). Reevaluating response and failure of medical treatment of endometriosis: a systematic review. Fertil Steril.

[248] Jenkins TR, Liu CY, White J (2008). Does Response to Hormonal Therapy Predict Presence or Absence of Endometriosis?. J Minim Invasive Gynecol.

[249] Bernuit D, Ebert AD, Halis G (2011). Female perspectives on endometriosis: Findings from the uterine bleeding and pain women’s research study. J Endometr.

[250] Casper RF (2017). Progestin-only pills may be a better first-line treatment for endometriosis than combined estrogen-progestin contraceptive pills. Fertil Steril.

[251] Vercellini P, Eskenazi B, Consonni D (2011). Oral contraceptives and risk of endometriosis: A systematic review and meta-analysis. Hum Reprod Update.

[252] Seracchioli R, Mabrouk M, Manuzzi L (2009). Post-operative use of oral contraceptive pills for prevention of anatomical relapse or symptom-recurrence after conservative surgery for endometriosis. Hum Reprod.

[253] Seracchioli R, Mabrouk M, Frascà C, Manuzzi L, Savelli L, Venturoli S (2010). Long-term oral contraceptive pills and postoperative pain management after laparoscopic excision of ovarian endometrioma: a randomized controlled trial. Fertil Steril.

[254] Takamura M, Koga K, Osuga Y (2009). Post-operative oral contraceptive use reduces the risk of ovarian endometrioma recurrence after laparoscopic excision. Hum Reprod.

[255] Wu L, Wu Q, Liu L (2013). Oral contraceptive pills for endometriosis after conservative surgery: a systematic review and meta-analysis. Gynecol Endocrinol Off J Int Soc Gynecol Endocrinol.

[256] Muzii L, Di Tucci C, Achilli C (2016). Continuous versus cyclic oral contraceptives after laparoscopic excision of ovarian endometriomas: A systematic review and metaanalysis. Am J Obstet Gynecol.

[257] Zorbas KA, Economopoulos KP, Vlahos NF (2015). Continuous versus cyclic oral contraceptives for the treatment of endometriosis: a systematic review. Arch Gynecol Obstet.

[258] Vercellini P, De Giorgi O, Mosconi P, Stellato G, Vicentini S, Crosignani PG (2002). Cyproterone acetate versus a continuous monophasic oral contraceptive in the treatment of recurrent pelvic pain after conservative surgery for symptomatic endometriosis. Fertil Steril.

[259] Zupi E, Marconi D, Sbracia M (2004). Add-back therapy in the treatment of endometriosis-associated pain. Fertil Steril.

[260] Chwalisz K, Perez MC, DeManno D, Winkel C, Schubert G, Elger W (2005). Selective Progesterone Receptor Modulator Development and Use in the Treatment of Leiomyomata and Endometriosis. Endocr Rev.

[261] Goenka L, George M, Sen M (2017). A peek into the drug development scenario of endometriosis – A systematic review. Biomed Pharmacother.

[262] Rocha ALL, Reis FM, Petraglia F (2012). New trends for the medical treatment of endometriosis. Expert Opin Investig Drugs.

[263] Islam MS, Afrin S, Jones SI SJ. Selective progesterone receptor modulators-mechanisms and therapeutic utility. Endocr Rev. 2020;41(5):bnaa012. PMID: 32365199. 10.1210/endrev/bnaa012 PMC8659360 32365199

[264] Bruner-Tran KL, Zhang Z, Eisenberg E, Winneker RC, Osteen KG (2006). Down-regulation of endometrial matrix metalloproteinase-3 and -7 expression *in vitro* and therapeutic regression of experimental endometriosis *in vivo* by a novel nonsteroidal progesterone receptor agonist, tanaproget. J Clin Endocrinol Metab.

[265] Kettel LM, Murphy AA, Mortola JF, Liu JH, Ulmann A, Yen SS (1991). Endocrine responses to long-term administration of the antiprogesterone RU486 in patients with pelvic endometriosis. Fertil Steril.

[266] Kettel LM, Murphy AA, Morales AJ, Ulmann A, Baulieu EE, Yen SS (1996). Treatment of endometriosis with the antiprogesterone mifepristone (RU486). Fertil Steril.

[267] Carbonell JL, Riverón AM, Leonard Y, González J, Heredia B SC. Mifepristone 2.5, 5, 10 mg versus placebo in the treatment of endometriosis. J Reprod Heal Med. 2016;217–25.

[268] ChwaliszC. Mattia-Goldberg K, Lee M, Elger W, Edmonds A. Treatment of endometriosis with the novel selective progesterone receptor modulator (SPRM) asoprisnil. Fertil Steril. 2004;82:S83–84. 10.1016/j.fertnstert.2004.07.212.

[269] Singh SS, Evans D, McDonald S, Senterman M, Strickland S (2020). Ulipristal Acetate Prior to Surgery for Endometriosis. Reprod Sci.

[270] Yao Z, Shen X, Capodanno I (2005). Validation of rat endometriosis model by using raloxifene as a positive control for the evaluation of novel SERM compounds. J Investig Surg Off J Acad Surg Res.

[271] Altintas D, Kokcu A, Kandemir B, Tosun M, Cetinkaya MB (2010). Comparison of the effects of raloxifene and anastrozole on experimental endometriosis. Eur J Obstet Gynecol Reprod Biol.

[272] Stratton P, Sinaii N, Segars J (2008). Return of chronic pelvic pain from endometriosis after raloxifene treatment: a randomized controlled trial. Obstet Gynecol.

[273] Kulak JJ, Fischer C, Komm B, Taylor HS (2011). Treatment with bazedoxifene, a selective estrogen receptor modulator, causes regression of endometriosis in a mouse model. Endocrinology.

[274] Harada T, Ohta I, Endo Y, Sunada H, Noma H, Taniguchi F (2017). SR-16234, a Novel Selective Estrogen Receptor Modulator for Pain Symptoms with Endometriosis: An Open-label Clinical Trial. Yonago Acta Med.

[275] Velasco I, Rueda J, Acién P (2006). Aromatase expression in endometriotic tissues and cell cultures of patients with endometriosis. Mol Hum Reprod.

[276] Rafique S, Decherney AH (2017). Medical Management of Endometriosis. Clin Obstet Gynecol.

[277] Ferrero S, Gillott DJ, Venturini PL, Remorgida V (2011). Use of aromatase inhibitors to treat endometriosis-related pain symptoms: a systematic review. Reprod Biol Endocrinol.

[278] Sibiude J, Santulli P, Marcellin L, Borghese B, Dousset B, Chapron C (2014). Association of history of surgery for endometriosis with severity of deeply infiltrating endometriosis. Obstet Gynecol.

[279] Goodman LR, Goldberg JM, Flyckt RL, Gupta M, Harwalker J, Falcone T. Effect of surgery on ovarian reserve in women with endometriomas, endometriosis and controls. Am J Obstet Gynecol. 2016. 10.1016/j.ajog.2016.05.029 10.1016/j.ajog.2016.05.029 27242204

[280] Leyland N, Casper R, Laberge P, Singh SS, SOGC. Endometriosis: diagnosis and management. J Obstet Gynaecol Can. 2010;32(7 Suppl 2):S1–32. http://www.ncbi.nlm.nih.gov/pubmed/21545757. 21545757

[281] Practice bulletin no (2010). 114: Management of endometriosis. Obstet Gynecol.

[282] Kuznetsov L, Dworzynski K, Davies M, Overton C. Diagnosis and management of endometriosis: summary of NICE guidance. BMJ. September 2017:j3935. 10.1136/bmj.j3935. 10.1136/bmj.j3935 28877898

